# Age-dependent stress response DNA demethylation and gene upregulation accompany nuclear and skeletal muscle remodeling following acute resistance-type exercise in rats

**DOI:** 10.1139/facets-2019-0060

**Published:** 2020-06-22

**Authors:** Erik P. Rader, Brent A. Baker

**Affiliations:** a Centers for Disease Control and Prevention, National Institute for Occupational Safety and Health, Morgantown, WV 26505, USA

**Keywords:** dorsiflexor muscles, dynamometer, Fischer 344XBrown Norway rats, stretch-shortening contractions, skeletal muscle, aging

## Abstract

Efficacy of high-intensity resistance exercise becomes progressively compromised with aging. Previously, to investigate this, we developed a rodent model of high-intensity training consisting of stretch-shortening contractions (SSCs) and determined that following one month of training, young rats exhibit a robust stress response and 20% performance increase, whereas old rats display a muted stress response and 30% performance decrease. Whether these age-specific responses occur early in training and constitute primary factors in adaptation/maladaptation was not addressed. The aim of the present study was to characterize performance, remodeling, and stress response transcriptional profile 6–120 h following acute SSC exposure. For young rats, the stress response pathway was highly regulated (≥20 differentially expressed genes at each time point) and was accompanied by robust DNA demethylation, tissue remodeling, and isometric torque recovery. For old rats, a muted transcriptional profile (13 and 2 differentially expressed genes at 6 and 120 h, respectively) coincided with deficiencies in demethylation, muscle remodeling, and torque recovery. These findings occurred in the context of heightened chronic levels of stress response gene expression with aging. This demonstrates that age-related constitutive elevations in stress response gene expression was accompanied by diminished SSC-induced responsiveness in epigenomic regulation and tissue remodeling.

## Introduction

Skeletal muscle, the most abundant tissue of the human body accounting for ~40% of total body mass, is critical for movement and metabolic homeostasis of glucose, lipid, and amino acids (Karagounis and Hawley 2010). After 50 years of age, skeletal muscle mass and performance decline at a rate of ~10% per decade (Offord and Withan 2017). High-intensity resistance exercise is promoted to enhance skeletal muscle mass and strength, yet outcomes for older individuals are typically blunted or compromised (Greig et al. 2011; Stewart et al. 2014; Stec et al. 2017). To investigate this muted responsiveness at old age, our laboratory employed an in vivo dynamometer model to expose muscles of rats to stretch-shortening contractions (SSCs) with each SSC consisting of an isometric, lengthening, and shortening contraction—the typical contraction sequence during traditional resistance exercise (Cutlip et al. 2006). This model permitted precise control of mode, velocity, excursion, and muscle activation while allowing whole muscle isolation for post-exposure analysis. Demanding training of 80 SSCs (8 sets of 10 repetitions) performed three sessions per week for one month induced 20% gains in muscle mass and performance for young 3-month-old rats (late adolescence or young adulthood) (Cutlip et al. 2006; Rader et al. 2016, 2017b; Rader and Baker 2017; Naimo et al. 2019). In contrast, muscles of old rats 30 months of age (comparable with 65–70 year old humans) maladapted to such training in that muscle mass did not hypertrophy and performance decreased by 30% (Cutlip et al. 2006; Rader et al. 2016, 2017b; Rader and Baker 2017; Naimo et al. 2019). This training-induced detrimental outcome highlighted the importance of characterizing potential underlying age-related molecular mechanisms.

The cellular stress response consists of universal mechanisms to cope with stress-induced damage to macromolecules including physiological mechanisms for sensing lipid, protein, and DNA damage; redox sensing and regulation; controlling cell cycle control; and stabilizing and repairing macromolecules (Galluzzi et al. 2018). Such a response is critical for establishing appropriate remodeling to stress. Resistance training consisting of high-intensity SSCs exposes muscle to mechanical, oxidative, and thermal stress. Studies in humans have indicated that an age-related perturbation of distinct heat shock proteins and redox homeostasis is present in the untrained state and thereby predicts a blunted stress response to exercise (Cobley et al. 2015). Consistent with this notion, previous research has established that with one month of such training, the muscles of young rats mounted a robust stress response consisting of heat shock proteins, antioxidants, and a gene expression profile consistent with cell cycle modulation (Murlasits et al. 2006; Ryan et al. 2010; Naimo et al. 2019). In contrast, muscles of old rats exhibited a muted response in these measures thereby implicating a deficient cellular stress response as contributing to age-related maladaptation to high-intensity SSCs (Murlasits et al. 2006; Ryan et al. 2010; Naimo et al. 2019).

Whether the characteristics of the stress response is an early factor in adaptation or maladaptation to high-intensity SSC training was left unresolved. The aim of the present study was to characterize age-dependent SSC-induced alterations in performance, tissue remodeling, and the stress response transcriptional profile 6–120 h following a single exposure to SSCs. The hypothesis tested is that at young age, SSC-induced stress response gene regulation is robustly sustained and concurrent with performance recovery and tissue remodeling, whereas at old age, such responses are blunted. Upon observing extensive gene upregulation for the stress response pathway exclusively for young rats, nucleus morphology, promoter DNA methylation, and growth arrest and DNA damage-inducible 45 alpha (GADD45α, a key stress sensor with cell cycle arrest, DNA repair, and DNA demethylation roles) were then investigated as potential influences on gene expression regulation (Tamura et al. 2012).

## Materials and methods

### Animals

Male Fischer Brown Norway hybrid rats (F344 × BN F1) were obtained from the National Institutes of Aging colony. Rats 3 and 30 months old were singly housed in an Association for Assessment and Accreditation of Laboratory Animal Care accredited animal quarters. Food and water were provided ad libitum. Temperature and light:dark cycle (dark cycle, 0700–1900) were held constant. The animals were acclimated to housing conditions for one week, segregated by age, and randomized to SSC groups with 6, 24, 48, 72, and 120 h recoveries (Fig. S1). After performance testing at each recovery time point while still anesthetized (isoflurane gas 2%–3% by volume), all animals were euthanized by pentobarbital (100–300 mg/kg body weight) intraperitoneal injection followed by exsanguination. All animal procedures were done in accordance to the Guide for the Care and Use of Laboratory Animals (8th edition, National Academies Press) and approved by the Animal Care and Use Committee at the National Institute for Occupational Safety and Health in Morgantown, West Virginia.

### SSC exposure

The SSC exposure was based on a previously described procedure to emulate a high-intensity training session at a relatively demanding volume of 8 sets of 10 repetitions for a muscle group (Cutlip et al. 2006). Each rat was anesthetized with isoflurane gas (2%–3% by volume), placed supine on a heated table, and the left knee was secured in flexion at 90° (Fig. S2). The left foot was secured to a fixture containing a load cell. Platinum electrodes were placed subcutaneously in the region of the common peroneal nerve for activation of the dorsiflexor muscles. Stimulation for all contractions was set at 4 V magnitude, 0.2 ms pulse duration, and 120 Hz frequency, optimal parameters for maximal contraction (Geronilla et al. 2003). Maximal isometric tetanic contraction was determined by activating for 300 ms with the ankle at 90°.

Exposure to SSCs consisted of eight sets with 2-min intervals between sets and 10 SSCs per set with 2-s intervals between SSCs to be comparable to the pauses between repetitions and the rest times between sets that are not uncommon during resistance training (Váczi et al. 2011). For each SSC, the dorsiflexor muscles were maximally activated while the ankle was set to 90° for 100 ms, then rotated to 140° at 60°/s, returned to 90° at the same velocity, and lastly, deactivated 300 ms later. The 60° per second velocity was chosen to promote muscle adaptation rather than overt muscle degeneration and injury as was found at a higher velocity (500° per second) in other studies (Cutlip et al. 2006; Baker et al. 2008, 2010). At 2 min after the SSCs, isometric torque was measured at the same settings as those for the maximum isometric tetanic contraction. Each rat was then returned to their cage and allowed to recover for either 6, 24, 48, 72, or 120 h at which time, maximum isometric tetanic torque was assessed and then compared with nonexposed values utilizing pre-SSC values. These time periods were chosen to diminish the influence of fatigue and based on prior work establishing that performance and morphological alterations become apparent 6–72 h following SSCs that then begin to resolve afterwards (Baker et al. 2006). Immediately following isometric torque assessment, both right and left tibialis anterior (TA) muscles were surgically removed, weighed, and the tibia lengths recorded. Normalized muscle mass was determined by dividing the muscle mass by tibia length and right TA muscles were utilized as nonexposed controls for muscle mass, morphology, gene expression, and DNA methylation analyses. Values for maximum isometric tetanic performance, muscle mass, and quantitative morphology for these groups of rats for the 6, 24, 48, 72, and 120 h time points were previously reported without reference to nonexposed control values or statistical analysis of post-SSC values expressed as percentages of nonexposed values (Baker et al. 2008). The present report is a secondary analysis of this data along with the addition of nonexposed control values for baseline reference and analysis of post-SSC values as expressed relative to nonexposed values so that magnitude of SSC-induced alterations could be assessed. Muscle quality was quantified as maximum isometric tetanic torque by normalized muscle mass.

### Quantitative morphology

The mid-belly of each TA muscle was covered with tissue freezing media and immersed in isopentane (−160 °C). This tissue was then cryosectioned at 12 μm thickness, hematoxylin and eosin stained, and analyzed by a standard stereological method (Baker et al. 2006; Rader et al. 2017b, 2018). At 2-mm offset from either side of the midpoint, five equally spaced fields (at 40× magnification) were evaluated. For each field, the points of an overlay graticule (121-point, 11-line grid) were analyzed as previously illustrated (Rader et al. 2017b). Since there were a total of 10 fields, 1210 points were evaluated. Each point was determined to be overlaying a nondegenerative muscle fiber, degenerative muscle fiber, cellular interstitium, or noncellular interstitium. Degenerative muscle fibers were defined as having (*i*) loss of contact with surrounding fibers, (*ii*) interdigitation of the sarcolemma by cellular infiltrates, and (*iii*) internalization of cellular infiltrates (Baker et al. 2006; Rader et al. 2018). Points overlaying interstitial nuclei were considered cellular interstitium, whereas points overlaying interstitial regions without nuclei were coded as noncellular interstitium. Percent of muscle tissue compromised of nondegenerative muscle fiber, degenerative muscle fiber, cellular interstitium, or noncellular interstitium were calculated as the percentage of points which overlaid each tissue type relative to total number of points.

### Gene expression analysis

A ~25-mg portion of frozen TA muscle tissue was homogenized with a Minin-BeadBeater 8 (Biospec) while in a vial of 1 mL of TRIzol with 1.0-mm zirconia beads (BioSpec, Bartlesville, OK, USA; Cat. #11079110zx). The RNAqueous phenol-free total RNA Isolation Kit (Thermo Fisher Scientific, Waltham, MA USA; Cat. #AM1912) was used to isolate RNA and cDNA was synthesized utilizing 0.3 μg of RNA and the RT^2^ First Strand Kit (Qiagen, Cat. #330401). Stress response and growth or remodeling relevant gene expression were evaluated by analyzing samples with two respective RT^2^ Profiler™ polymerase chain reaction (PCR) Arrays (Qiagen, Germantown, MD, USA; Cat. #PARN-012Z and Qiagen, Cat. #PARN-058Z) per manufacturer’s instructions. The analysis template provided by Qiagen (PCRArrayDataanalysis_V4) was used to determine fold changes and *p*-values for cycle threshold (*C*_*t*_) data. Genes were considered differentially expressed when fold regulation exceeded 1.3-fold regulation (below 0.769-fold change or above 1.3-fold change) and *p* < 0.05 (Rader et al. 2017a). Gene expression fold change across all the differentially expressed stress response genes was averaged to determine overall gene expression of the stress response pathway. Bioinformatic analysis of differentially expressed genes was performed by Ingenuity Pathway Analysis (IPA; Ingenuity Systems, ingenuity.com). IPA was used to determined activation *z*-scores, a statistical measure of the match between (*i*) expected gene expression for a given functional outcome based on literature with (*ii*) the observed gene expression of the present study. This allows for prediction on whether distinct biological functions are activated. IPA assigns significance when activation *z*-scores > 2 or < −2.

### Immunofluorescence

The mid-belly of each TA muscle was transversely cryosectioned at 12 μm thickness. Sections were fixed in HistoChoice (Sigma-Aldrich, St. Louis, MO, USA; H2904) for 45 min, washed (3 × 5 min in phosphate-buffered saline), permeabilized (0.4% Triton X-100 for 10 min), washed (3 × 5 min in PBS), and then blocked with 10% goat serum in phoshate-buffered saline with 0.1% Triton X-100 (PBST) at room temperature for 1 h. A primary polyclonal antibody for growth arrest and DNA damage-inducible 45 alpha, GADD45α (Biorbyt; orb135380; 1:40) was applied overnight at 4 °C. Sections were washed (3 × 5 min in PBS) and secondary antibody (goat anti-rabbit IgG Alexa Fluor 488 at 1:500 in PBST) was applied for 2 h. After 3 × 5 min washes in PBS, sections were mounted with Prolong™ Gold Antifade Reagent (Thermo Fisher Scientific; P36931) with 4′,6-diamidino-2-phenylindole (DAPI). For each muscle section, with the investigator blinded to sample identification, 10 images were captured at site locations determined in the same manner as described for quantitative morphology. Image analysis utilized ImageJ (version 1.46, National Institutes of Health, USA) for evaluation and treating the images for each section as two dimensional. For analysis of total area labeled for GADD45α as a percentage of muscle section area and particle analysis regarding nuclei, threshold was set based on secondary only images. Particle analysis of the DAPI image yielded nuceli size and circularity. The index of circularity was determined by the equation 4*π* (area/perimeter^2^) with a perfect circle as a value of 1 and increasing elongation as the value decreases. Colocalization analysis was performed using the ImageJ plugin Colocalization_class which was utilized for determination of nuclear staining for GADD45α thereby labeling colocalized regions as light yellow (Rader et al. 2018).

### DNA methylation

Total DNA was purified from a ~25 mg portion of frozen TA muscle utilizing the DNeasy Blood & Tissue Kit (Qiagen, Cat. #69506) and gene promoter DNA methylation was assessed utilizing a stress response relevant EpiTect Methyl II PCR Array (Qiagen, Cat. #EARN-121Z) per manufacturer’s instructions. The principle of this system was based on PCR quantification of remaining DNA following either digestion of unmethylated DNA, methylated DNA, both unmethylated and methylated DNA, or a mock digestion (i.e., no enzymes included) of DNA. A quantity of 1 μg of genomic DNA was added to the restriction digestion buffer to make a final volume of 120 μL, which was then partitioned to the four different restriction digest conditions and incubated for 6 h at 37 °C. Each digest was then added to a PCR master mix and then loaded (25 μL) into each well of the array in which each well contained primers flanking a distinct promoter region of interest (the array was designed for 22 genes). The PCR was then run utilizing an Applied Biosystems 7500 Real-Time PCR (Thermo Fisher Scientific) instrument. *C*_*t*_ values were entered into an analysis template provided by Qiagen for EARN-121Z to calculate percent methylation. For each group, data for all of the genes included for the array (with the exceptions of *Apaf1* and *Bad* that did not pass quality control) were averaged to represent the overall effect on the stress response pathway. To calculate fold change, percent methylation for each muscle was divided by mean percent methylation of the appropriate control group.

### Statistical analysis

Data were analyzed using ANOVA with the variable of animal identification as a random factor to account for repeated measures within animal when appropriate. Post-hoc comparisons were performed using Fisher’s least significant difference method. All data were shown as means ± error in which error was standard error of the mean with the exception of stress response pathway gene expression fold change data in which error was defined as fold change × ln2 × sqrt ($\sigma_{x}^{2}/n_{x}\;+\;\sigma_{y}^{2}/n_{y}$) per manufacturer’s instructions (GeneGlobe Data Analysis Center, Qiagen); *p* < 0.05 was considered statistically significant.

## Results

Several age-related muscular declines were apparent by 30 months of age in the old rats. Old rats exhibited sarcopenia in the form of diminished normalized muscle mass (Table 1). Maximum isometric torque capacity and muscle quality were also decreased with age by 17% and 14%, respectively (Table 1). These age-related decrements were accompanied by alterations in quantitative morphology.

A decrease in percentage tissue composition of nondegenerative muscle fibers (92.9% ± 0.4% vs. 95.1% ± 0.3%, *N* = 29–30 per group, *p* < 0.0001) was observed at old age (Fig. S3A and S3B). This decrease was accounted for by an increased tissue percentage of interstitium, specifically, noncellular interstitium with age—6.2% ± 0.4% vs. 4.2% ± 0.2%, *N* = 29–30 per group, *p* < 0.0001 (Fig. S2A and S2B), which has previously been reported to be representative of increased fibrosis or collagen (Rader et al. 2015).

**Fig. 1.**
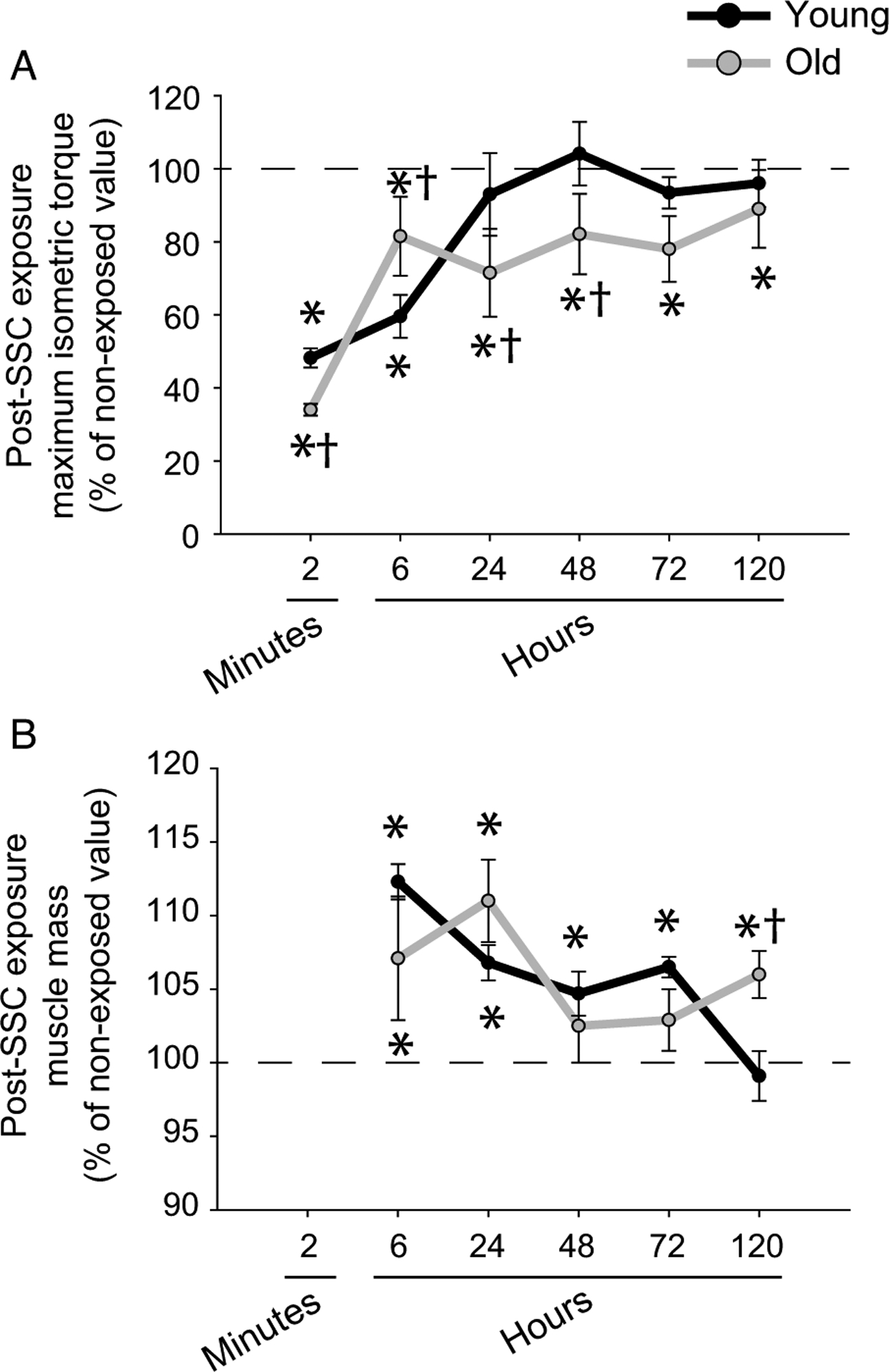
Compromised recovery of muscle performance and mass following a single stretch-shortening contraction (SSC) exposure was observed for old rats. (A) Dorsiflexor isometric torque outputs for young rats was approximately half of nonexposed (i.e., pre-SSC exposed) values at 2 min and 6 h post-SSC exposure and then recovered to nonexposed values by 1 d. For old rats, torque output at 2 min was more severe than that of young rats but rose rapidly to 80% of nonexposed values at 6 h. However, values of old rats did not reach nonexposed values even by 5 d. (B) Tibialis anterior (TA) muscle mass values (post-SSC absolute values expressed as percentage of nonexposed contralateral values) for young rats were elevated following SSC exposure and returned to nonexposed values by 5 d. For old rats, muscle mass did not return to nonexposed values at 5 d. For 2 min post-SSC exposure, sample sizes were *N* = 30 per group. For the remaining time points post-SSC exposure, sample sizes were *N* = 6 per group. *, value for SSC-exposed muscles distinct from nonexposed value; †, value for old different from value from young, *p* < 0.05.

As previously shown, compromised recovery in muscle mass and performance was evident with aging following SSCs (Baker et al. 2008). Muscles of young rats recovered isometric torque output by 24 h and muscle mass returned to nonexposed values by 120 h (Figs. 1A and 1B). Relative to muscles of young rats after SSC exposure, muscles of old rats exhibited a greater isometric torque deficit at 2 min. This outcome for old rats was indicative of a heightened fatigue response apparent at 2 min. The higher early fatigue suggested that muscle tension was depressed for old rats during the SSC exposure and thereby may have limited exposure to damaging high contractile tension. Consistent with this notion, the torque deficit was diminished for old rats at 6 h (when muscle is further recovered from fatigue and torque better reflects the sustained condition of the muscle). Despite the diminished torque deficit at 6 h of old relative to young rats, performance and muscle mass of old rats did not return to control values by the last time point investigated, 120 h (Figs. 1A and 1B).

An age-related decrease in tissue remodeling post-SSC exposure was observed by quantitative morphology. For muscles of young rats, a small increase in tissue percentage of degenerative muscle fibers was observed 72 h after the SSCs, 1.116% ± 0.513% for SSC-exposed muscles at 72 h vs. 0.003% ± 0.003% for nonexposed muscles, *N* = 6–29 per group, *p* < 0.0001 (Fig. S3I). This was accompanied by evidence of an increased tissue composition of interstitium: 1.8% ± 0.3% vs. 0.7% ± 0.1% (*N* = 6–29 per group, *p* < 0.0001) for cellular interstitium, and 5.4% ± 0.8% vs. 4.2% ± 0.2% (*N* = 6–29 per group, *p* = 0.0834, trend) for noncellular interstitium (Fig. S3I). For muscles of old rats, cellular interstitium increased following SSCs, 1.4% ± 0.3% at 72 h vs. 0.9% ± 0.1% for nonexposed muscle, *N* = 6–30 per group, *p* = 0.0110 (Fig. S3J). However, no increases in tissue percentages of degenerative muscle fibers or noncellular interstitium were evident at any time points following the SSC exposure for old rats (Fig. S3). Therefore, quantitative morphology analysis demonstrated that the age-related compromise in recovery observed in this study was in the context of diminished tissue remodeling in the days following SSC exposure.

### Differential gene expression relevant to growth or remodeling and stress response were muted at old age

**Fig. 2.**
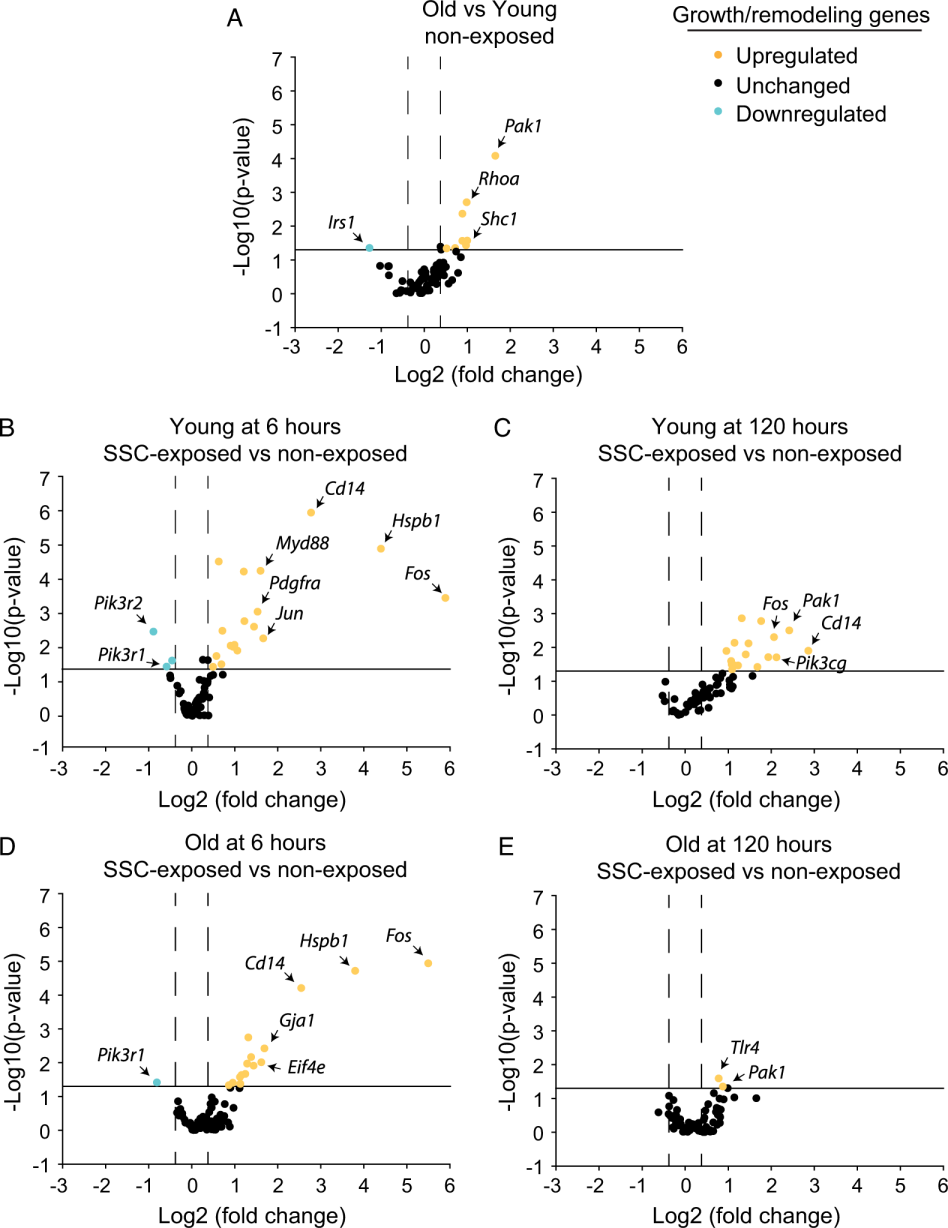
An age-related deficiency in recovery post stretch-shortening contraction (SSC)-exposure was reflected in muted gene expression for growth and remodeling relevant genes especially at 120 h. Differential gene expression was determined for (A) nonexposed muscles of old vs. young rats and SSC-exposed vs. nonexposed muscles of (B) young rats at 6 h, (C) young rats at 120 h, (D) old rats at 6 h, and (E) old rats at 120 h. Sample sizes were *N* = 6 per group. The solid line denotes a *p*-value cut-off of 0.05 and dashed lines represent 1.3-fold regulation for determination of differential gene expression. For each group, the most extreme differentially expressed genes were identified. Refer to Table S1 for more detailed information.

**Fig. 3.**
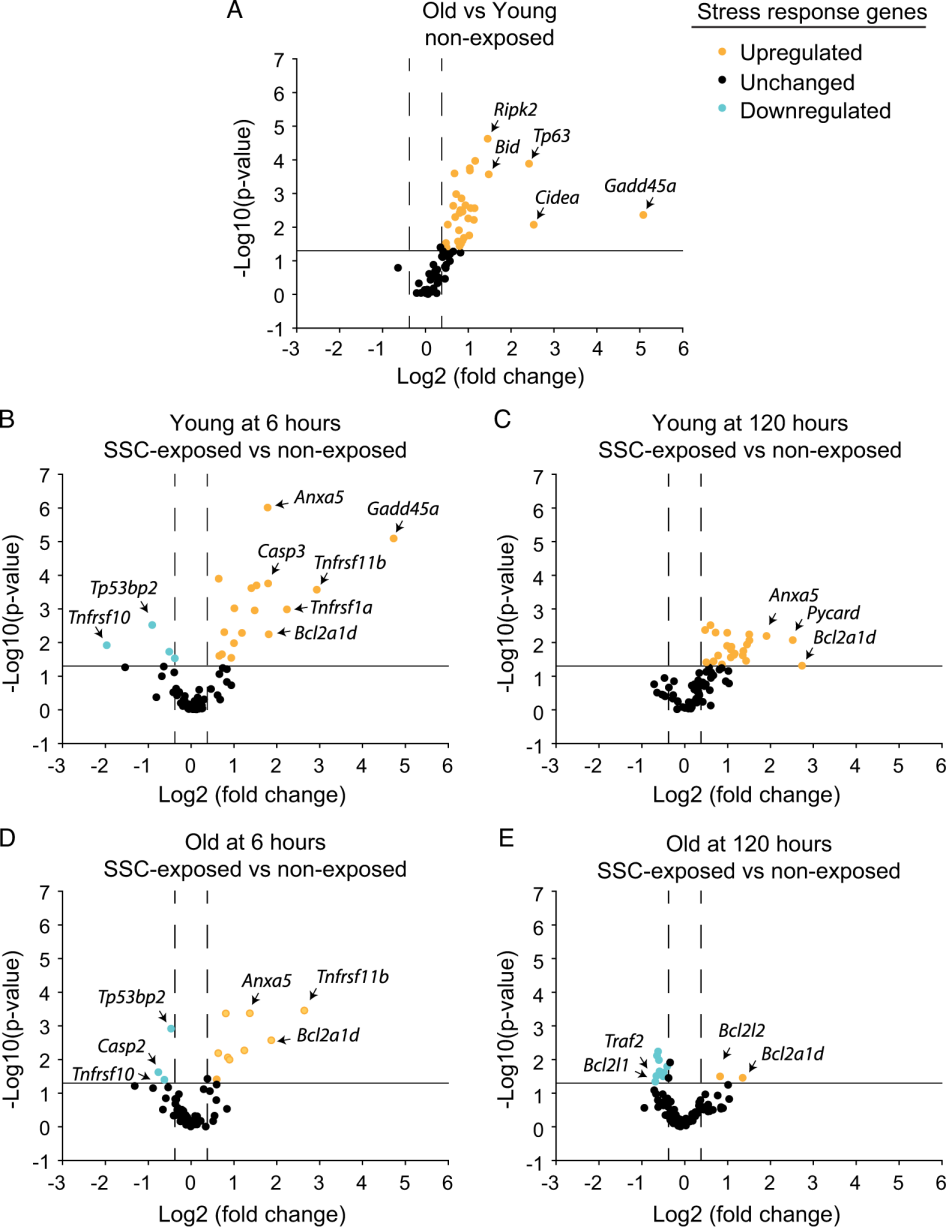
Compromised recovery or remodeling at old age was accompanied by a muted gene expression in stress response genes. Differential gene expression was determined for (A) nonexposed muscles of old vs. young rats and stretch-shortening contraction (SSC)-exposed vs. nonexposed muscles of (B) young rats at 6 h, (C) young rats at 120 h, (D) old rats at 6 h, and (E) old rats at 120 h. Sample sizes were *N* = 3–6 per group. The solid line denotes a *p*-value cut-off of 0.05 and dashed lines represent 1.3-fold regulation for determination of differential gene expression. For each group, the most extreme differentially expressed genes were identified. Refer to Table S2 for more detailed information.

Gene expression for growth or remodeling and stress response relevant genes were predominantly elevated with aging (Figs. 2A and 3A; Tables S1 and S2). When collectively, differential gene expression is analyzed via IPA, a profile of chronic inflammatory signaling coupled with anabolic resistance emerges. Consistent with chronic inflammatory signaling with aging, IPA predicted upstream activation of the cytokines tumor necrosis factor (TNF) (*p* = 6.83E-07 and activation *z*-score = 3.080) and interferon gamma (*p* = 2.03E-07 and activation *z*-score = 2.960) and activation of the biological functions of leukocyte migration (*p* = 1.57E-06 and activation *z*-score = 2.376) and cell movement of leukocytes (*p* = 1.17E-05 and activation *z*-score = 2.008). The activation of these biological functions was based on the upregulation of *Pak1*, *Ripk2*, *Pycard*, *Abl1*, *Rhoa*, *Tp53*, *Tnfrsf1a*, *Akt1*, *Pik3r1*, and *Sos1*. Furthermore, the process of fibrogenesis (*p* = 1.50E-07 and activation *z*-score = 1.352) was consistent with upregulation of *Pak1*, *Pycard*, *Abl1*, *Rhoa*, *Tnfrsf1a*, and *Akt1*. Meanwhile, *Irs1* was downregulated and IPA network analysis predicted decreased activation of the PI3K complex, both findings indicative of anabolic resistance and the observed age-related decrease in muscle mass. Characteristic with the inflammatory signaling and an overall activation of stress pathways associated with aging, *Gadd45a* (a major stress sensor with roles in cell cycle arrest, DNA repair, cell survival, and apoptosis) was the most highly differentially expressed gene with a 34-fold difference (Fig. 3A and Table S2) (Tamura et al. 2012).

To investigate the gene expression profiles at the onset of age-related differences following SSCs and at a later time period corresponding with more enduring long-term differences, analysis was focused at both the earliest and latest experimental time points (i.e., 6 and 120 h) at which muscle tissue was collected (Figs. 2B–2E and 3B–3E; Tables S1 and S2). For young rats at 6 h, large fold changes in the expression of distinct growth or remodeling growth genes were observed. However, growth and remodeling as a whole was likely not optimal at this time period since IPA network analysis predicted inactivation of the PI3K complex based on the downregulation of *Pik3r1* and *Pik3r2*. Meanwhile, stress response gene pathway expression was robust with 16 upregulated genes. IPA denoted upstream activation of various cytokines (TNF, IL15, PRL, IL174, CSF1, and IL1β, *p*-values < 0.0001 and activation *z*-scores > 2.0) and activation of inflammatory signaling—mononuclear leukocyte (*p* = 5.86E-09 and activation *z*-score = 2.180) and T cell (*p* = 1.47E-07 and activation *z*-score = 2.408) responses based on upregulation of *Cd14*, *Tnfrsf1a*, *Casp3*, *Myd88*, *Tnfrsf1b*, *Pycard*, and *Pik3r1*. Consistent with gene upregulation marking the onset of stress related signaling, *Gadd45a* was upregulated by 26-fold (Fig. 3B and Table S2).

At 120 h for young rats, stress relevant genes continued to be differentially expressed with 22 upregulated genes. Interestingly, *Gadd45a* upregulation was 2.6-fold relative to nonexposed value, whereas growth and remodeling pathways were upregulated (Fig. 3C; Tables S1 and S2). IPA indicated that cytokine (e.g., CSF1, PRL, IL17A, IFNγ, and IL1β) and inflammatory signaling remained active in the categories of leukocyte recruitment, migration, and accumulation (*p* < 0.0001 and activation *z*-scores > 2.0) based on upregulation of *Pycard*, *Pak1*, *Pik3cg*, *Fos*, *Prkcb*, *Tlr4*, *Casp1*, *Itgb1*, *Abl1*, *Tnfrsf1a*, *Myd88*, *Tnfsf12*, and *Ltbr*. Unique to the 120 h time period was a gene expression profile consistent with growth promotion. IPA denoted biological functions of DNA interaction, angiogenesis, and neuritogenesis (*p* < 0.0001 and activation *z*-scores > 2.0) largely based on upregulation of such genes as *Igf1*, *Itgb1*, *Ilk*, and *Pik3cg*. Furthermore, *Pabpc1*, a facilitator of protein translation, was upregulated 3.2-fold and IPA network analysis predicted that the PI3K family was active.

At old age for both the 6 and 120 h time periods, *Gadd45a* was not upregulated and differential expression of genes for stress response and growth/remodeling were muted (Figs. 2D, 2E, 3D, and 3E; Tables S1 and S2). The most extreme blunting of gene expression response was apparent at 120 h. Only 11 stress relevant genes were differentially expressed, nine of which were downregulated. Furthermore, only two growth- and remodeling-related genes were differentially expressed with no augmentation to *Pabpc1* expression. Due to this limited expression profile, IPA did not predict any biological functions or upstream regulators.

### Altered nucleus size and shape following SSCs were age dependent

**Fig. 4.**
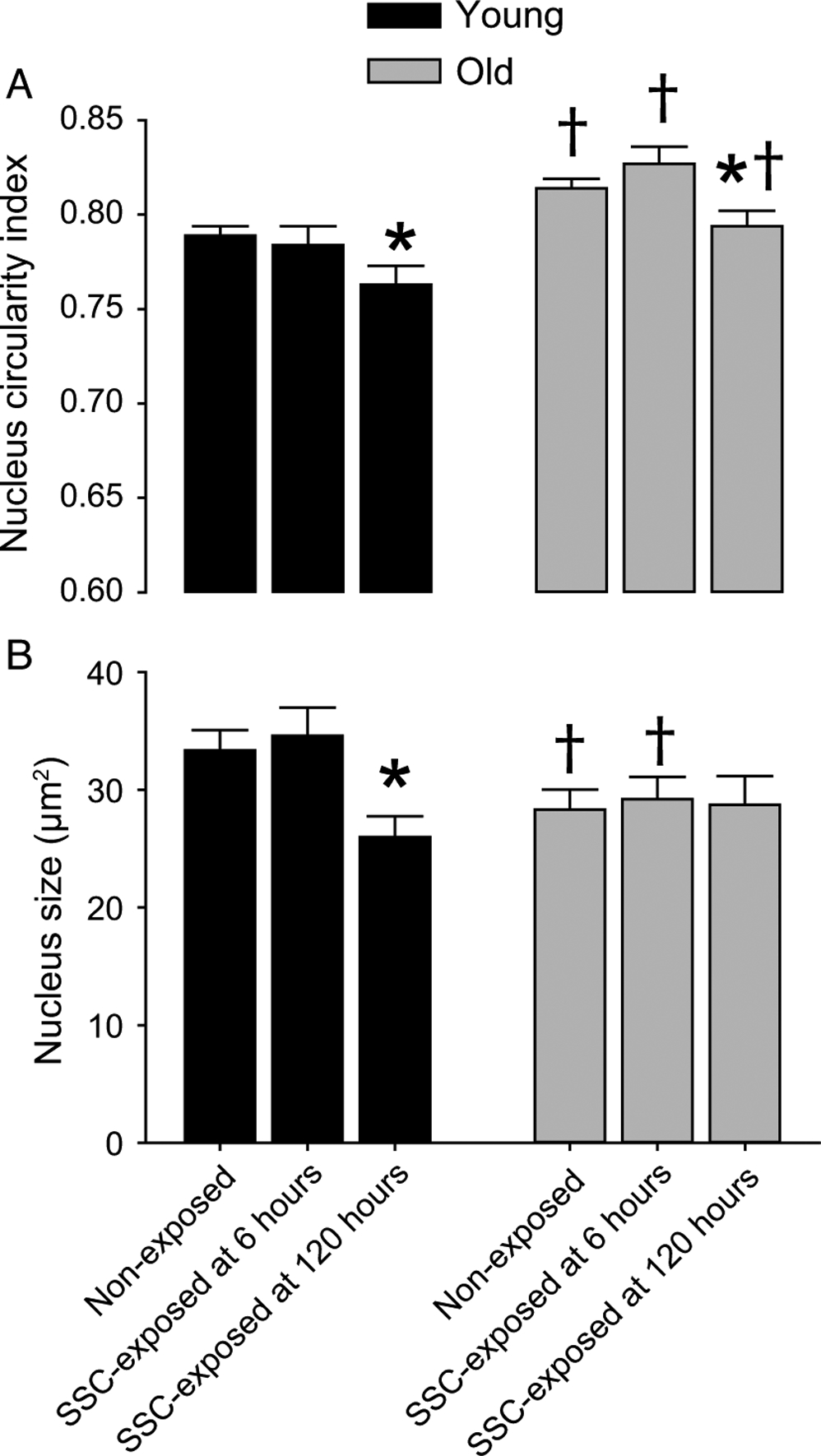
Nucleus size and shape following stretch-shortening contractions (SSCs) were age dependent. (A) Nucleus circularity index denotes a perfect circle at a value of 1 and increasing elongation at lower values. (B) Nucleus size was determined by evaluating particle size (i.e., area) of 4′,6-diamidino-2-phenylindole staining (refer to Fig. S1 for representative images). Sample sizes were *N* = 5–11 muscles per group (with 217 ± 4 nuclei evaluated per muscle). *, value for SSC-exposed muscles distinct from non-exposed value; †, value for old different from value from young*, p* < 0.05.

Decreased nuclear size and elongated nuclear shape are features that typically accompany DNA condensation and less accessibility for transcription (Mascetti et al. 2001; Versaevel et al. 2012). To investigate whether these features coincided with age-dependent differences in gene expression, nuclear morphology was assessed by immunofluorescence (Figs. 4 and S4). For nonexposed muscles, the density of total nuclei increased with age (old vs. young, 996 ± 23 vs. 826 ± 26 nuclei per mm^2^, *N* = 8–11 per group, *p* < 0.0001). While the notion of increased nuclei density with aging is debated because of divergent findings in the literature likely because of variation in methodology (Larsson et al. 2019), the result agrees with our previous work regarding rats and has been reported by other groups (Gallegly et al. 2004; Malatesta et al. 2009; Naimo et al. 2019). Interestingly, the nuclei morphology responded in a divergent manner in terms of transcriptional accessibility with nuclei becoming smaller yet more circular with age (Figs. 4A and 4B). Only the more circular morphology is consistent with increased transcriptional accessibility and the increased transcriptional regulation observed for old vs. young nonexposed muscles. For muscles of young rats, SSC exposure induced decreased nuclear circularity (thereby, denoting nuclear elongation) and smaller size specifically at 120 h (Figs. 4A and 4B). No SSC-induced changes in density of total nuclei number were observed (nonexposed, 6 h, and 120 h—826 ± 26, 850 ± 38, and 892 ± 23 nuclei per mm^2^, *N* = 5–8 per group). For old rats, with the exception of nuclear circularity, SSCs did not induce alterations in any nuclei metrics which were assessed (Figs. 4A and 4B). Nuclear circularity decreased at 120 h post-SSCs for muscles of old rats but values did not reach young levels (Fig. 4A). These results were consistent with age-specific responsiveness in nucleus morphology alterations accompanying less extreme fold increases in gene upregulation at 120 h relative to 6 h post-SSC exposure.

### DNA demethylation of the stress response pathway was observed exclusively at 120 h for young rats

**Fig. 5.**
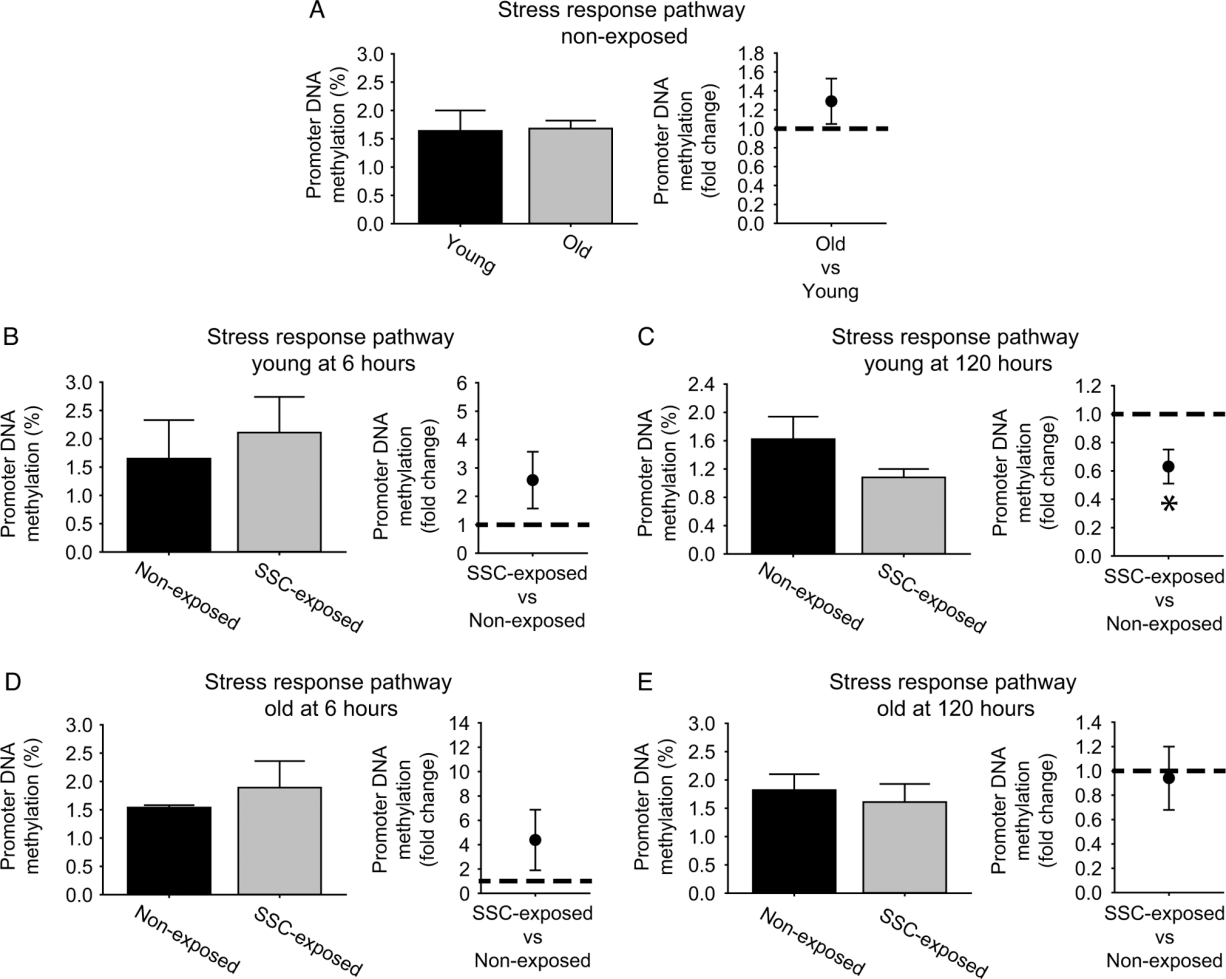
Overall promoter DNA demethylation was observed for the cellular stress response pathway exclusively for stretch-shortening contraction (SSC) exposed young muscles at 120 h. Percent methylation of promoter regions for various stress response genes were determined for (A) young and old nonexposed muscles, (B) young rats at 6 h post-SSC exposure, (C) young rats at 120 h post exposure, (D) old rats at 6 h post SSC exposure, and (E) old rats at 120 h post-SSC exposure. Sample sizes were *N* = 4–12 per group. Dashed line denotes an equal degree of methylation between groups. *, fold change was significant, *p* < 0.05.

While nuclear elongation and decreased nuclear size coincided with less extreme gene upregulation at 120 h, these morphological changes would not explain the persistent moderate upregulation for a large set of genes we observed at young age in the stress response pathway. To resolve this, DNA demethylation was investigated as an alternative mechanism for increased gene expression (Fig. 5; Tables S3, S4, and S5). For both age groups at 6 h, no fold changes in methylation at the overall stress response pathway level were observed (Figs. 5B and 5D). Yet, at 6 h, gene expression for the overall cellular stress response increased by 2.2 ± 0.4 fold for young rats and 1.7 ± 0.2 fold for old rats relative to non-exposed controls, *N* = 3–6 per group, *p* < 0.05, thereby demonstrating the possibility of earlier methylation alterations which were not evaluated in the present study or methylation-independent gene expression regulation at this time point. Both early DNA methylation modulation and gene expression regulation in the absence of methylation alterations are not uncommon occurrences (Barres et al. 2012; Turner et al. 2019). The expected inverse relationship between promoter DNA methylation and gene expression was observed at the stress response pathway level for young rats at 120 h. Mean percentage DNA methylation across the cellular stress response genes was decreased at 0.6 ± 0.2 fold values of nonexposed muscles (with six genes displaying differential methylation) and gene expression of the overall stress response remained elevated at a 2.3 ± 1.1 fold increase, *N* = 3–6 per group, *p* < 0.05 (Fig. 5C). Meanwhile, no SSC-induced modulations in overall cellular stress pathway methylation and gene regulation (0.8 ± 0.1 fold change, *N* = 3–6 per group, *p* > 0.05) were observed (Fig. 5E). Since GADD45α has been reported as a mediator of demethylation as well as a central stress sensor, tissue distribution of GADD45α was analyzed by immunofluorescence to provide supporting data regarding SSC-induced demethylation (Barreto et al. 2007; Voisin et al. 2015). For nonexposed muscles, an age-related increase in in baseline GADD45α distribution was observed supportive of the role of GADD45α as a stress marker. For young rats at 6 h post-SSCs, total GADD45α distribution trended for an increase and, more importantly, GADD45α staining area within each nuclei significantly increased by 1.4-fold, changes coinciding with 26-fold *Gadd45a* upregulation (Fig. 3; Figs. S4 and S5). Consistent with the muted methylation alterations observed for old rats, SSCs did not induce an alteration in GADD45α distribution at old age (Fig. S5).

## Discussion

SSCs consisting of a consecutive sequence of isometric, lengthening, and shortening contractions are common during daily movements and high-intensity resistance training (Váczi et al. 2011). Our research has routinely been focused on investigating such SSCs directly rather than isometric, lengthening, or shortening contractions in isolation to ensure studies are applicable to this common contraction modality (Cutlip et al. 2006; Baker et al. 2008; Rader et al. 2016, 2017b; Naimo et al. 2019). The major finding of the present work was the characterization of an age-specific response to an acute exposure of high-intensity SSCs. By 6 h, skeletal muscles of young rats differentially expressed 20 stress response genes, whereas old rats differentially expressed 13 genes. By 120 h for young rats, the stress response pathway exhibited DNA demethylation and continued upregulation of stress response genes (22 genes). Meanwhile for old rats, blunted stress response pathway demethylation was observed and a limited stress response upregulation (two genes) was induced. Furthermore for old rats, a muted tissue remodeling response was noted at both the transcriptional and histological level accompanied by compromised muscle recovery of muscle mass and performance. These results occurred in the context of chronically high expression of stress response genes at baseline for nonexposed muscles at old age (30 genes were upregulated, and no genes were downregulated for old vs. young nonexposed muscles). These results provide insight into a blunted stress response to SSCs and the early events of susceptibility to maladaptation from high-intensity training with aging.

Bioinformatics regarding differentially expressed growth or remodeling genes predicted PI3K anabolic signaling at 120 h and not at 6 h post-SSCs for young rats of the present study. Meanwhile, *Gadd45a* was highly upregulated (26-fold) and GADD45α tissue distribution was heightened at 6 h thereby suggesting an emphasis on stress response at this early time period. *Gadd45a* expression is induced by DNA damage from physiological and environmental stressors and is considered an influential stress sensor mediating cell cycle arrest, differentiation, DNA repair, or apoptosis (Tamura et al. 2012; Bullard et al. 2016). Our finding of an early *Gadd45a* upregulation was consistent with animal and human studies regarding acute exposure to lengthening contractions (Chen et al. 2002, 2003). These reports interpreted contraction-induced *Gadd45a* upregulation as an adaptive response with emphasis on the role of GADD45α in cell cycle modulation and DNA repair. Specifically, the investigators proposed that GADD45α was essential for maintaining muscle fibers in a differentiated state (preventing gap 1 to synthesis cell cycle transition) while in the context of an otherwise pro-growth and pro-proliferative transcriptional upregulation. Thereby, *Gadd45a* upregulation for young rats in the present study potentially maintained the post-mitotic state of muscle fibers during muscle adaptation and remodeling. Other cell types within muscle tissue, specifically those with proliferative capacity (e.g., satellite cells, endothelial cells, and interstitial cells), could have also benefited from cell-cycle withdrawal in that delaying entry into synthesis and mitotic phase would allow more time for DNA repair prior to proliferation and growth. Interestingly, GADD45α also has a role in mediating active DNA demethylation (Rai et al. 2008; Niehrs and Schafer 2012). GADD45α-mediated DNA demethylation does not appear to be global but rather to be gene-specific in mammalian cells (e.g., *MLH1* and *MMP-9*) (Engel et al. 2009; Schafer et al. 2010; Zhou et al. 2018). Therefore, *Gadd45a* upregulation at the 6-h time point for young rats may have had an influential role at both allowing DNA repair and then establishing demethylation allowing for continued gene expression at the later 120-h time point.

Decreased nuclear size and elongated nuclear shape typically accompany DNA condensation and less accessibility for transcription (Mascetti et al. 2001; Versaevel et al. 2012). DNA damage induces a global chromatin condensation response believed to be a protective mechanism against further damage (Hamilton et al. 2011). This decreased accessibility would be consistent with the decrease in extreme gene expression fold changes observed at 120 h versus 6 h for young rats. However, DNA condensation would not have explained the continued moderate upregulation of many of the genes including those in the stress response pathway evaluated at 120 h. Rather, this may be partly attributable to the observation of promoter demethylation regarding the stress response pathway at this time period since promoter demethylation generally correlates positively with transcription (Bird 2002; Jjingo et al. 2012). Considering that GADD45α-mediated DNA demethylation appears to be gene specific rather than global in mammalian cells, such a specific modulation may have orchestrated the targeted response of the young rats in the present study—with demethylation contributing to upregulation of the stress response pathway specific for young rats at 120 h while in the context of nuclear shape changes indicative of global transcriptional repression (Engel et al. 2009; Schafer et al. 2010). Overall, our data are suggestive of an appealing hypothesis—one in which nuclear or chromatin remodeling imparts global protection of DNA while concurrent demethylation permits upregulation of specific key response genes and, ultimately, pathways. Consistent with this notion, data exclusive for young rats at 120 h in the present study, hypomethylation paralleled the downregulation of the stress response pathway all in the framework of a nuclei elongation and nuclei size decrease. A limitation of the present study was that stress response DNA demethylation induced regulation of gene expression was not tested by direct manipulation of DNA methylation status of distinct genes. Rather, the results establish parallel instances of stress response DNA methylation and gene expression for young rats following SSC exposure. Indeed, the causal link between this DNA methylation and gene expression needs to be investigated further in future work.

The muscles of old rats in the present study lacked SSC-induced nucleus size alterations, robust stress response pathway DNA demethylation, and persistent gene upregulation while functional recovery was compromised. This indicates that at old age, the coordination between the stress response for cell-cycle arrest, DNA damage, and GADD45α-mediated demethylation with global nuclear or chromatin compaction was absent. Such a lack of response would be expected to compromise muscle remodeling and recovery. This work greatly expanded upon prior research which focused on evaluating developmental myosin heavy chain labeling following acute SSC exposure (Baker et al. 2008). Muscles of old rats displayed blunted developmental myosin heavy chain labeling following SSCs thereby suggesting an age-related limitation in tissue remodeling as a factor for reduced recovery (Baker et al. 2008). While the present data regarding remodeling or growth transcription were consistent with this previous research, the current finding of an age-related impairment in stress response demethylation and gene expression as potentially underlying the response is novel.

This lack of SSC-induced stress response occurred in the context of a chronic age-related upregulation of stress response genes. Upregulation of stress response genes including *Gadd45a* with aging alone was not surprising given that DNA damage accumulates with age (Ryan et al. 2008, 2010). Such age-related constitutive elevation of the stress response transcription and *Gadd45a* may suggest that acute regulation of these genes would be compromised. Chronic elevated levels of a response pathway underlying a lack of acute sensitivity has been observed before in a different context—specifically, a muted inflammatory response followed a muscle strain injury in old rats which exhibit chronic inflammatory signaling (Rader et al. 2015; Rader and Baker 2017). Therefore, such a phenomenon has potential as a general framework in which to view blunted responses to stress with aging.

The muted expression of stress response and growth or remodeling genes along with the compromised tissue remodeling (in regards to the absence of robust alterations in quantitative morphology and nucleus size) of old rats in the present study implies that muscles at old age would not be as well prepared for exposure to subsequent bouts of SSCs. Indeed, frequent SSC exposure results in skeletal muscle maladaptation with aging (Cutlip et al. 2006; Rader et al. 2016, 2017b; Rader and Baker 2017; Naimo et al. 2019). Furthermore, the finding of a lack of SSC-induced stress response pathway DNA demethylation at old age (unlike the young) suggests that this pathway is not as accessible for transcriptional activity upon further SSC training. Therefore, the overall data were consistent with the notion that too frequent high-intensity training is detrimental at old age (Cutlip et al. 2006; Rader et al. 2016, 2017b; Rader and Baker 2017; Naimo et al. 2019). Yet, previous research regarding old rats demonstrates adaptive responses in anabolic signaling, hypertrophy, and performance following less frequent high-intensity training (Rader et al. 2017b; Naimo et al. 2019). Therefore, optimizing the duration between less frequent SSC training bouts were sufficient for adaptation despite reliance on a more limited remodeling capacity with aging. When considering this past work with the present study, the implication is that modulation of parameters for high-intensity SSC training (e.g., lowering training frequency) is necessitated at old age as a means to compensate for compromised responsiveness in features such as stress response gene expression and tissue remodeling.

## Supplementary Material

supplemental figures

## Figures and Tables

**Table 1.** Muscle mass and performance values for all nonexposed muscles for young and old rats utilized in the study.

	Young rat	Old rat
Body weight (g)	326 ± 5	575 ± 8*
Muscle mass/body weight (mg/g)	1.85 ± 0.01	1.12 ± 0.02*
Tibial length (mm)	40.3 ± 0.2	44.7 ± 0.1*
Normalized muscle mass (mg/mm)	15.0 ± 0.2	14.3 ± 0.2*
Maximum isometric torque (mN-m)	74.2 ± 2.1	61.1 ± 2.3*
Muscle quality (mN-m/mg/mm)	5.0 ± 0.1	4.3 ± 0.2*

**Note:** Sample sizes were *N* = 30 per group.

* , value for old different from value for young, *p* < 0.05.
