# Supplementary material for: Age-dependent stress response DNA demethylation and gene upregulation accompany nuclear and skeletal muscle remodeling following acute resistance-type exercise in rats
Source: Facets (Ott). Author manuscript; Available in PMC 2020 Aug 7. (PMC7413608; doi:10.1139/facets-2019-0060)
Supplement: supplemental figures [file NIHMS1607941-supplement-supplemental_figures.pdf]

Maximal isometric tetanus assessed and SSC protocol administered to left dorsiflexors of young and old rats.

N = 30 per age group

↓

Maximal isometric tetanus assessed for left dorsiflexors at either 6, 24, 48, 72, or 120 hours post SSC exposure. At each time point, right (non-exposed) and left (SSC-exposed) tibialis anterior muscles removed for analysis.

N = 6 per age group at each time point

Figure S1. Study design

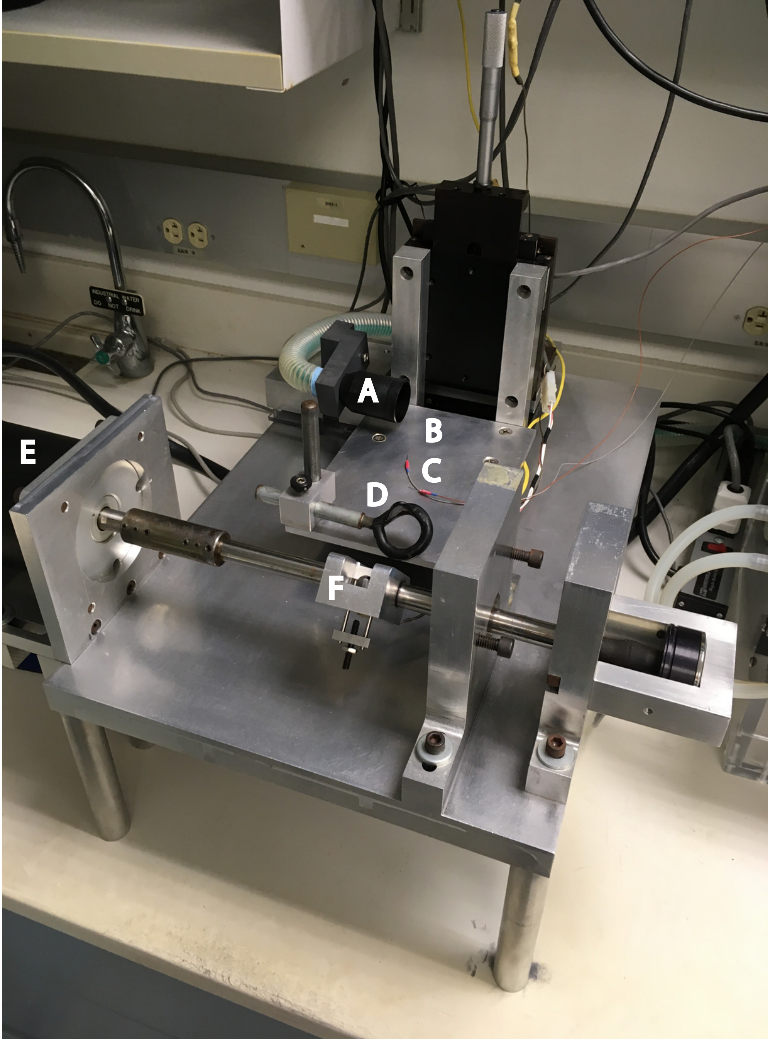

Figure S2. Apparatus for SSC exposure. (A) Anesthesia nose cone, (B) heated table, (C) platnium needle electrodes, (D) knee holder, (E) servomotor and (F) fixture with load cell to secure foot are noted.

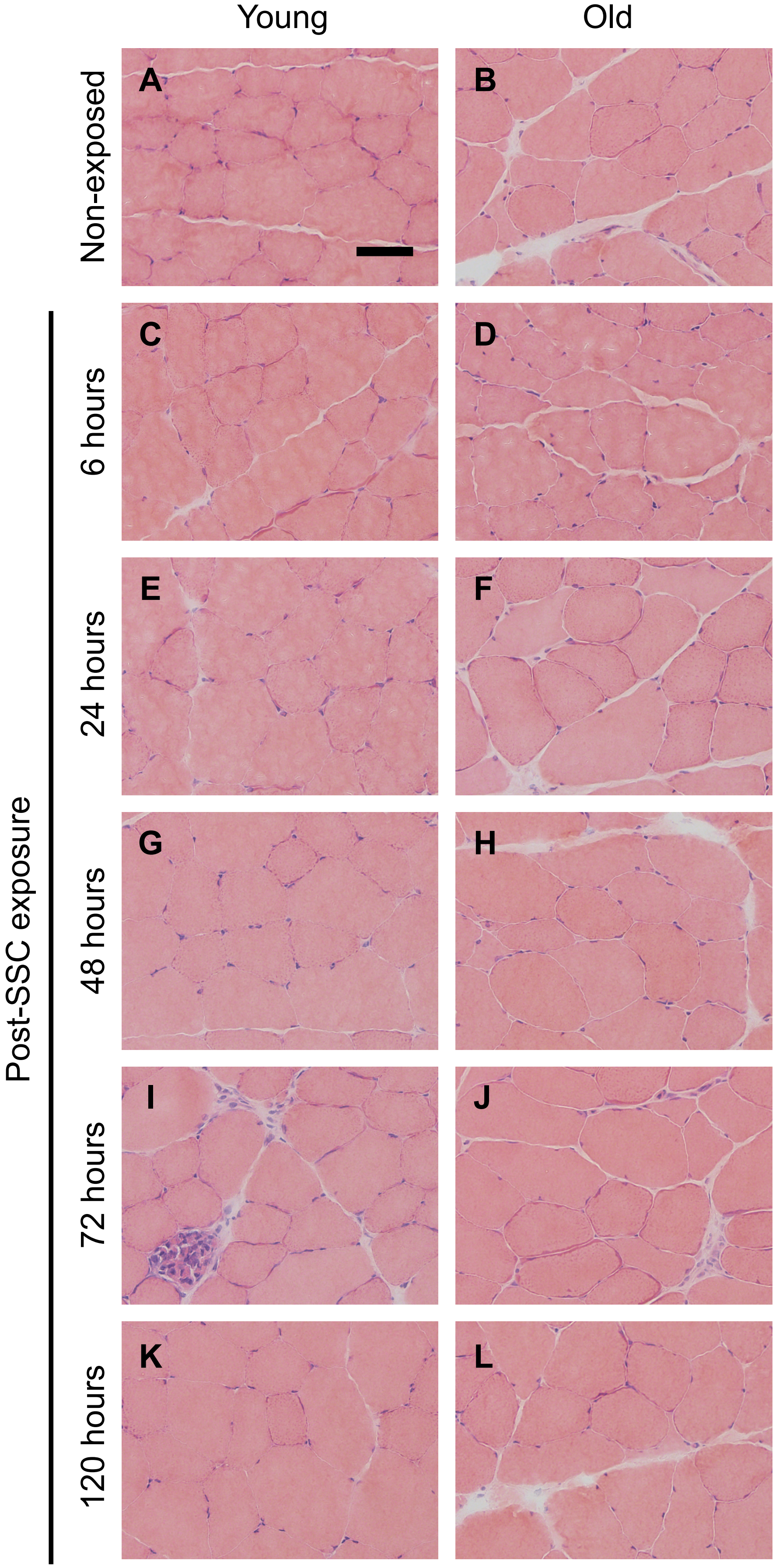

Figure S3. Hematoxylin and eosin stained muscle sections from non-exposed (A,B) and SSC-exposed muscles (C-L) of young and old rats. 40x magnification. Scale bar = 50 µm.

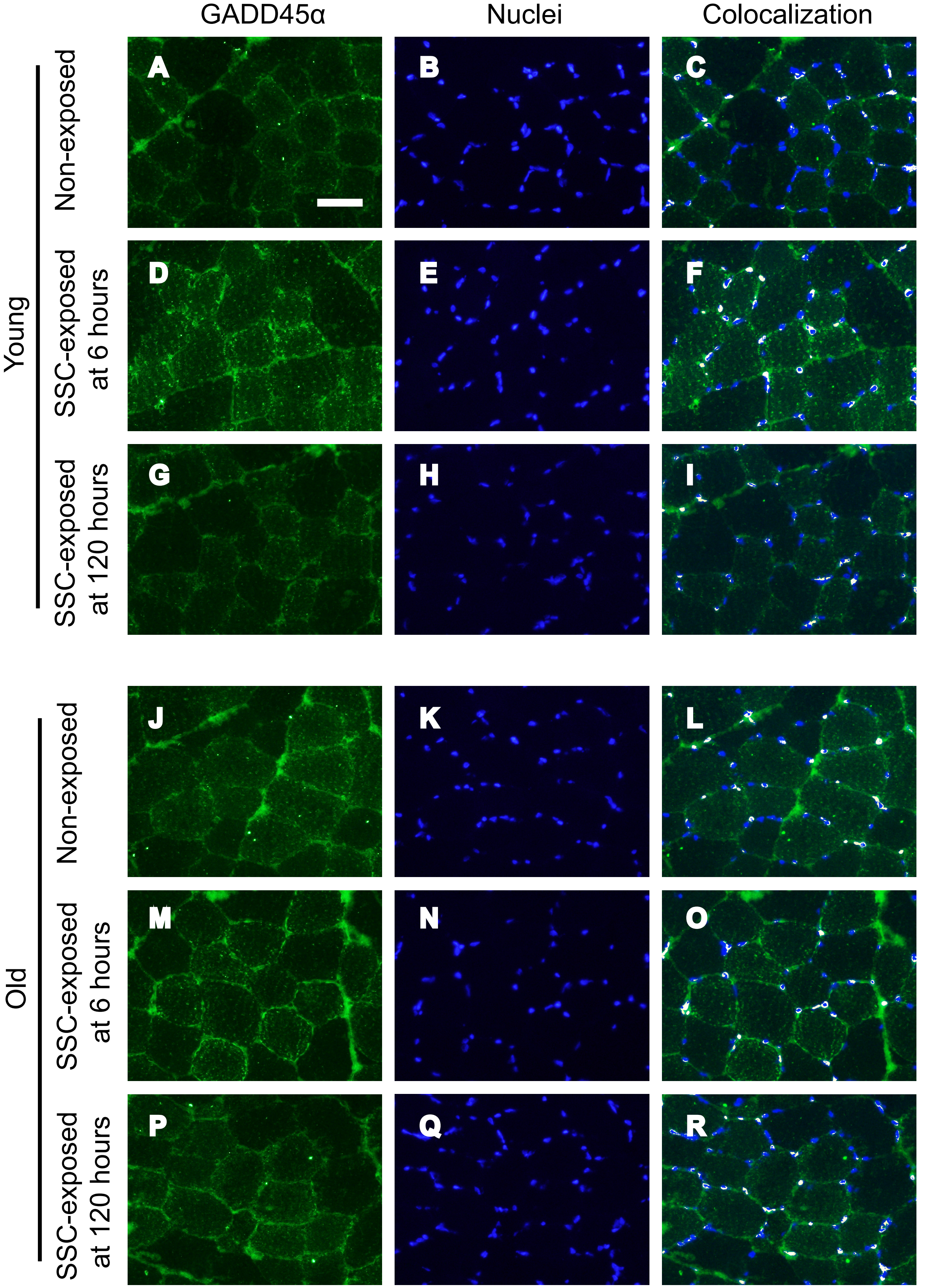

Figure S4. GADD45α (green) and nuclei (blue) were identified by immunofluorescence. Colocalization (light yellow) was determined utilizing ImageJ. Images depicted are for non-exposed and SSC-exposed muscles of young (A-I) and old (J-R) rats. 40x magnification. Scale bar = 50 µm.

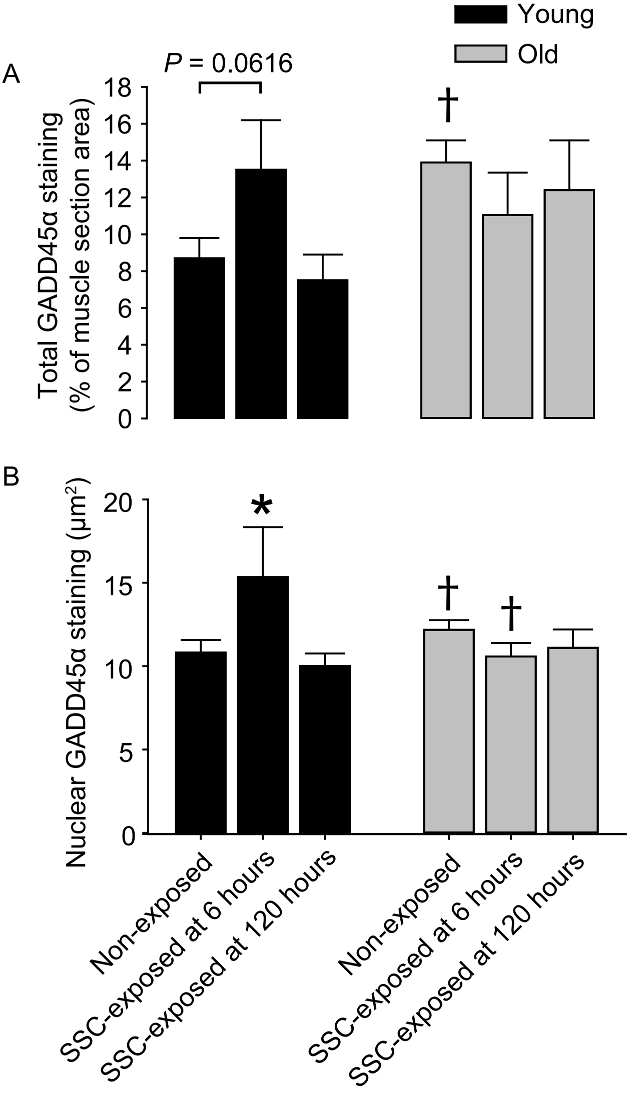

Figure S5. Increased area of nuclear GADD45α immunostaining at 6 hours post SSC exposure exclusively at young age. (A) Total GADD45α staining was determined by assessing the GADD45α distribution over the entire muscle section area evaluated. (B) Nuclear GADD45α staining represents the typical particle size (in terms of area) of colocalized GADD45α and DAPI staining. Sample sizes were N = 5 to 11 per group. *Value for SSC-exposed muscles distinct from non-exposed value, †Value for old different from value from young, $P < 0.05$.

		Non-exposed old v young		SSC-exposed young vs non-exposed young				SSC-exposed old vs non-exposed old			
				6 hours		120 hours		6 hours		120 hours	
	RefSeq	Fold change	P value	Fold change	P value	Fold change	P value	Fold change	P value	Fold change	P value
Adar	NM_031006	1.015	0.3495863	1.213	0.2140539	2.129	0.0451882	1.591	0.3496899	1.680	0.2651865
Akt1	NM_033230	1.029	0.3156168	1.127	0.3472970	1.312	0.2608956	1.600	0.7981331	1.200	0.7938643
Akt2	NM_017093	0.800	0.4636373	1.177	0.2729961	1.152	0.4216094	1.846	0.7849050	1.573	0.9314246
Akt3	NM_031575	0.753	0.8450449	1.014	0.7957626	2.165	0.0744891	1.432	0.6410061	1.693	0.3076758
Apc	NM_012499	0.675	0.9422761	1.947	0.0095230	1.332	0.2827003	3.073	0.0097984	1.673	0.6062752
Bad	NM_022698	1.021	0.2312555	1.032	0.8933062	1.944	0.0128060	1.617	0.7935037	1.486	0.8943071
Btk	NM_001007798	1.088	0.8068535	1.309	0.9634360	2.965	0.0699993	1.940	0.0394869	1.643	0.2523489
Casp9	NM_031632	1.219	0.5196473	1.001	0.6711187	2.114	0.0318192	1.356	0.5889438	1.860	0.1071210
Ccnd1	NM_171992	0.704	0.4264880	2.334	0.0016842	1.823	0.0593948	2.175	0.0269519	1.660	0.1915375
Cd14	NM_021744	1.376	0.2886580	6.840	0.0000011	7.281	0.0126005	5.842	0.0000621	2.209	0.0939831
Cdc42	NM_171994	1.019	0.2710694	0.948	0.5879353	1.492	0.3067583	1.556	0.6737300	1.279	0.9412692
Cdkn1b	NM_031762	0.640	0.9715605	0.706	0.0654720	1.274	0.2683920	1.222	0.4880503	1.496	0.9688465
Chuk	NM_001107588	0.802	0.9270856	1.230	0.1485784	1.346	0.3096390	1.539	0.4506987	1.403	0.7080145
Csnk2a1	NM_053824	0.835	0.6351494	0.989	0.8006326	1.162	0.4269725	1.378	0.9460552	1.375	0.9708412
Ctnnb1	NM_053357	0.813	0.8051577	1.288	0.1097575	1.167	0.3788654	1.559	0.4936785	1.242	0.9028892
Eif2ak2	NM_019335	0.945	0.4022980	1.007	0.8688934	2.038	0.0947184	1.385	0.9175409	1.572	0.5150396
Eif4b	NM_001008324	1.015	0.3158502	1.110	0.3425637	1.343	0.2020257	1.432	0.8927313	1.300	0.8988173
Eif4e	NM_053974	0.688	0.7929727	1.640	0.0032265	1.253	0.2785900	2.710	0.0123119	1.752	0.5341397
Eif4ebp1	NM_053857	0.967	0.3548784	0.967	0.7058059	1.157	0.4846034	1.613	0.5612211	0.828	0.1124903
Eif4g1	XM_213569	0.697	0.8048950	1.190	0.3170623	0.931	0.9524830	1.466	0.9669034	1.501	0.9035472
Faslg	NM_012908	0.906	0.6396818	1.083	0.7293083	1.459	0.6127891	2.371	0.0217117	1.775	0.2160576
Fkbp1a	NM_013102	0.999	0.1908073	1.215	0.0945746	1.776	0.2270288	1.799	0.5922017	1.419	0.7679125
Fos	NM_022197	1.130	0.4292572	59.513	0.0003550	4.189	0.0049911	45.132	0.0000115	3.149	0.0993781
Foxg1	NM_012560	0.906	0.6396818	1.083	0.7293083	1.279	0.7215567	2.189	0.0427560	1.348	0.4356419
Foxo3	NM_001106395	0.689	0.8591063	1.613	0.0307167	1.119	0.4790210	1.636	0.5474143	1.219	0.7466365
Gja1	NM_012567	0.566	0.2868805	2.892	0.0008940	2.215	0.0073391	3.231	0.0038150	1.752	0.3483811
Grb10	NM_001109093	0.491	0.1512955	1.293	0.0965195	1.179	0.2215028	1.961	0.2184294	1.756	0.3598697
Grb2	NM_030846	0.866	0.5744967	1.197	0.0227951	1.449	0.1746431	1.690	0.3823727	1.337	0.9957924
Gsk3b	NM_032080	0.922	0.4583695	1.488	0.0177783	1.489	0.1612928	2.232	0.0232965	1.381	0.7903769
Hras	NM_001098241	0.560	0.1563668	0.939	0.5327527	0.842	0.3375776	1.502	0.4539323	1.229	0.8266386
Hspb1	NM_031970	1.960	0.0372317	21.060	0.0000129	2.488	0.0013772	13.920	0.0000192	0.878	0.2202804
Igf1	NM_178866	0.566	0.1536754	1.010	0.9836749	2.653	0.0162585	1.334	0.6490396	1.718	0.2046425
Igf1r	NM_052807	0.892	0.5795476	1.092	0.5843022	1.647	0.0777363	1.433	0.9647770	1.450	0.9268054
Ilk	NM_133409	0.959	0.2293459	1.088	0.3595490	2.217	0.0343196	1.419	0.9307417	1.499	0.8740400
Irak1	NM_001127555	1.221	0.1428188	1.067	0.4267540	1.411	0.2073787	1.275	0.9628969	1.062	0.5220114
Irs1	NM_012969	0.414	0.0445857	0.977	0.8222718	0.727	0.1040792	1.475	0.7121904	1.374	0.9971753
Itgb1	NM_017022	0.893	0.7046180	1.870	0.0088448	2.344	0.0344661	2.602	0.0068666	1.741	0.1018678
Jun	NM_021835	1.804	0.0837010	3.168	0.0053881	1.649	0.2329077	2.443	0.0107815	1.211	0.7577129
Kcnh8	NM_145095	0.906	0.6396818	1.083	0.7293083	1.238	0.7475094	2.189	0.0427560	1.348	0.4356419
Map2k1	NM_031643	1.256	0.2284148	1.359	0.0673604	1.027	0.4911515	1.702	0.1684608	0.931	0.5745796
Mapk1	NM_053842	1.163	0.3241351	0.793	0.1303443	1.203	0.4122577	1.151	0.8729717	1.057	0.8867484
Mapk14	NM_031020	0.839	0.6537029	0.899	0.7384171	0.694	0.2686090	1.191	0.7309275	0.923	0.4151131
Mapk3	NM_017347	1.346	0.2034205	0.903	0.7140912	1.761	0.0813878	1.064	0.9867548	1.126	0.7547294
Mapk8	XM_341399	1.045	0.5629154	1.649	0.0620716	1.444	0.2544273	1.371	0.5460163	1.079	0.8761154
Mtcp1	XM_001070795	1.046	0.6706387	0.729	0.0239590	0.720	0.3918778	0.858	0.3192227	1.073	0.6051408
Mtor	NM_019906	1.362	0.1206530	0.916	0.8712264	1.302	0.3085324	1.008	0.7654862	0.907	0.4675876
Myd88	NM_198130	1.251	0.3110766	3.032	0.0000573	2.099	0.0253565	2.488	0.0018026	1.072	0.8594360
Nfkb1	XM_342346	1.140	0.4497781	2.085	0.0122810	1.103	0.4254764	1.819	0.0468586	0.966	0.9731361
Nfkbia	NM_001105720	1.418	0.1621948	1.326	0.2872309	1.691	0.1220222	1.019	0.9709138	1.037	0.7083479
Pabpc1	NM_134353	1.848	0.0273988	2.313	0.0000601	3.208	0.0380829	1.326	0.2919038	1.151	0.5931786
Pak1	NM_017198	3.146	0.0000837	1.130	0.2873416	5.353	0.0031378	0.903	0.6015207	1.836	0.0448578
Pdgfra	NM_012802	0.964	0.9636219	2.728	0.0024529	2.775	0.0076434	1.864	0.0559812	1.364	0.3728045
Pdk1	NM_053826	1.055	0.5830082	0.888	0.6161716	1.054	0.5685395	1.030	0.9886646	0.976	0.9330220
Pdk2	NM_030872	1.006	0.8291810	0.878	0.4414970	0.830	0.7350327	1.094	0.6614284	0.805	0.3799998
Pdpk1	NM_031081	1.337	0.1756123	1.408	0.0365262	1.234	0.3852514	1.380	0.2147575	0.868	0.3922856
Pik3ca	NM_133399	1.213	0.2900169	1.412	0.0629710	1.364	0.2434653	1.354	0.1674438	1.042	0.9797867
Pik3cg	NM_001106723	1.569	0.3989113	1.127	0.9340903	4.358	0.0196743	1.451	0.1423597	1.588	0.0703731
Pik3r1	NM_013005	1.644	0.0448065	0.668	0.0359715	1.437	0.2640307	0.571	0.0387254	0.905	0.6348700
Pik3r2	NM_022185	1.017	0.7332350	0.540	0.0034340	1.179	0.3140043	0.815	0.2555928	1.081	0.8683149
Prkca	NM_001105713	0.892	0.5489238	1.040	0.6295525	0.899	0.9929308	1.013	0.9546605	0.859	0.4234501
Prkcb	NM_012713	1.481	0.5058358	1.226	0.9554543	3.817	0.0195367	1.380	0.1071964	1.983	0.0500448
Prkcz	NM_022507	1.672	0.0571137	0.934	0.5136579	0.872	0.8635523	1.082	0.8344702	1.120	0.5762354
Pten	NM_031606	1.283	0.1217087	0.902	0.6367995	1.381	0.2185142	0.823	0.2506749	0.981	0.9681199
Ptk2	NM_013081	1.305	0.1387679	0.929	0.9261132	1.247	0.2675518	0.861	0.3904491	0.841	0.3813059
Ptpn11	NM_013088	0.883	0.5882587	1.100	0.3372715	0.812	0.7813010	1.063	0.8445279	0.835	0.3260876
Rac1	NM_134366	1.299	0.0410298	1.136	0.0876750	1.179	0.3638345	0.962	0.6802820	0.771	0.0839062
Raf1	NM_012639	1.227	0.1873535	1.053	0.6111353	1.144	0.4299225	0.969	0.6887957	0.864	0.3145820
Rasa1	NM_013135	1.229	0.1679196	1.029	0.6845863	1.660	0.1301727	1.059	0.7300314	1.079	0.6487772
Rbl2	NM_031094	1.170	0.1921933	0.710	0.0785628	1.296	0.2522845	0.803	0.1394871	0.986	0.9582353
Rheb	NM_013216	1.314	0.0500674	1.079	0.3872607	1.161	0.4381648	1.010	0.9677144	0.785	0.3267656
Rhoa	NM_057132	1.981	0.0019808	1.295	0.0233357	2.114	0.1654996	1.106	0.5659846	0.844	0.4014363
Rps6ka1	NM_031107	1.725	0.2439544	1.140	0.9541408	2.058	0.1535088	1.096	0.7926545	1.387	0.2296831
Rps6kb1	NM_031985	1.167	0.2245281	0.946	0.4926042	1.431	0.2423899	0.816	0.3707313	0.921	0.6337674
Shc1	NM_053517	1.999	0.0270380	0.887	0.5957245	2.056	0.1258262	0.891	0.4866495	0.911	0.5829368
Sos1	NM_001100716	1.434	0.0459079	0.990	0.9457741	1.297	0.3099740	0.834	0.2369358	0.862	0.5189351
Srf	NM_001109302	0.950	0.9627303	1.073	0.4520754	1.004	0.8246521	1.069	0.8855601	1.018	0.9255043
Tcl1a	NM_001109601	0.906	0.6396818	1.083	0.7293083	1.238	0.7475094	2.189	0.0427560	1.348	0.4356419
Tirap	XM_001055833	1.027	0.7100951	0.958	0.7970259	1.162	0.3902702	0.997	0.8158010	1.242	0.5342038
Tlr4	NM_019178	1.058	0.8155293	1.155	0.5503378	3.403	0.0016578	1.200	0.3978932	1.717	0.0257253
Tollip	NM_001109668	1.233	0.3364909	1.991	0.0083446	1.099	0.3826275	2.162	0.0570199	1.031	0.7986860
Tsc1	NM_021854	0.935	0.9654909	1.091	0.5008206	1.323	0.1257079	1.265	0.4074346	1.449	0.1485628
Tsc2	NM_012680	1.294	0.1658883	0.818	0.2194138	1.309	0.3045317	0.977	0.6055270	0.773	0.1735883
Wasl	NM_001110365	1.126	0.4675927	0.839	0.1886795	1.192	0.3179934	0.790	0.3047494	0.765	0.2919149
Ywhah	NM_013052	1.849	0.0043249	1.545	0.0000307	2.153	0.1440412	1.148	0.4524425	0.914	0.5451459

Table S1. Differential expression of growth and remodeling relevant genes for muscles for old relative to young rats at baseline and for each age group following SSC exposure relative to non-exposed muscles. Expression which surpassed 1.3-fold regulation (below 0.769 fold change or above 1.3 fold change) with a P value < 0.05 were considered differentially expressed; orange – increased expression, blue – decreased expression. Sample sizes were N = 6 per group.

		Non-exposed old v young		SSC-exposed young vs non-exposed young				SSC-exposed old vs non-exposed old			
				6 hours		120 hours		6 hours		120 hours	
	RefSeq	Fold change	P value	Fold change	P value	Fold change	P value	Fold change	P value	Fold change	P value
Abl1	NM_001100850	2.031	0.0177477	1.186	0.7295228	2.244	0.0215134	0.825	0.3657595	0.709	0.2154338
Aifm1	NM_031356	1.792	0.0013938	0.869	0.6427884	1.174	0.5747515	1.065	0.4809139	0.838	0.7110971
Akt1	NM_033230	1.693	0.0268518	1.022	0.9295767	1.153	0.5904114	0.827	0.3623520	0.628	0.1052727
Anxa5	NM_013132	1.306	0.0745319	3.452	0.0000010	3.758	0.0064663	2.583	0.0004240	1.088	0.6953660
Apaf1	NM_023979	1.457	0.0583125	0.825	0.2987440	1.481	0.0552714	0.828	0.4664720	1.021	0.7307932
Api5	NM_001127379	1.759	0.0031277	1.110	0.7878462	1.376	0.1992333	0.909	0.4650617	0.659	0.0104310
Aven	NM_001107757	1.212	0.1846125	1.071	0.7434076	1.233	0.2592970	1.012	0.8912326	0.756	0.1438748
Bad	NM_022698	1.740	0.0038161	0.953	0.9207381	1.988	0.0051930	0.915	0.4492341	0.942	0.6253378
Bag1	NM_001106647	1.998	0.0055575	1.076	0.5339545	1.173	0.1754192	0.963	0.7626300	0.661	0.1792170
Bak1	NM_053812	1.862	0.0209772	2.000	0.0105576	2.219	0.0235043	1.064	0.8526216	0.828	0.4982403
Bax	NM_017059	2.188	0.0061274	1.670	0.0577202	1.754	0.0643708	0.871	0.5146831	0.763	0.2529597
Bcl10	NM_031328	1.486	0.1025074	2.274	0.0052386	1.825	0.0449628	1.513	0.0390027	0.844	0.3104047
Bcl2	NM_016993	1.063	0.8875873	1.091	0.9816859	1.104	0.9277011	1.168	0.6703366	1.361	0.3098574
Bcl2a1d	NM_133416	1.373	0.3454260	3.514	0.0057437	6.668	0.0492813	3.661	0.0026835	2.551	0.0351819
Bcl2l1	NM_031535	2.080	0.0027187	1.379	0.2424814	1.588	0.0950983	0.915	0.6069811	0.622	0.0458886
Bcl2l11	NM_022612	1.103	0.8426040	1.600	0.4979579	0.639	0.3114931	1.267	0.9882336	1.139	0.4177509
Bcl2l2	NM_021850	1.200	0.9236909	0.570	0.4261813	1.522	0.7536859	0.665	0.1422809	1.771	0.0319606
Bid	NM_022684	2.784	0.0002698	1.916	0.0288683	2.848	0.0087131	0.860	0.3114485	1.395	0.1091426
Bik	NM_053704	1.103	0.8426040	0.976	0.8519256	0.639	0.3114931	0.953	0.7106299	1.184	0.4112400
Birc2	NM_021752	1.435	0.0084601	2.795	0.0011112	1.262	0.1971361	1.754	0.0004277	0.650	0.0058271
Birc3	NM_023987	2.213	0.0027338	2.648	0.0002437	1.984	0.0126729	1.811	0.0087381	1.019	0.5719615
Birc5	NM_022274	1.103	0.8426040	0.976	0.8519256	0.693	0.3547614	0.953	0.7106299	1.127	0.4193053
Bnip2	NM_001106835	1.395	0.0299183	0.993	0.9167782	1.053	0.8358938	0.882	0.3376587	0.666	0.0225505
Bnip3	NM_053420	1.407	0.0401083	1.032	0.9669121	1.204	0.4713471	0.981	0.8549101	0.753	0.0161760
Bok	NM_017312	1.026	0.7940138	1.189	0.7411083	2.026	0.0707746	0.754	0.4669167	1.493	0.3449364
Card10	NM_001130554	0.987	0.7394952	1.005	0.9256599	0.895	0.5785815	0.801	0.3146368	1.377	0.2579542
Casp1	NM_012762	1.765	0.0577559	1.783	0.1495159	2.695	0.0353331	1.511	0.0559720	1.265	0.1891190
Casp12	NM_130422	1.645	0.0010469	0.703	0.0188978	1.595	0.0365874	0.809	0.2784627	1.289	0.1618769
Casp14	XM_234878	1.103	0.8426040	0.976	0.8519256	0.639	0.3114931	0.953	0.7106299	1.127	0.4193053
Casp2	NM_022522	1.193	0.2858350	0.643	0.0521231	1.531	0.0775077	0.589	0.0239317	1.186	0.3684708
Casp3	NM_012922	1.401	0.1310393	3.477	0.0001768	2.554	0.0243746	2.365	0.0053906	1.137	0.6675562
Casp4	NM_053736	1.600	0.0002537	0.978	0.7891731	1.817	0.0578560	0.922	0.8740963	0.994	0.7970816
Casp6	NM_031775	1.148	0.3027243	0.979	0.8362135	2.840	0.0057881	0.872	0.5375216	1.460	0.2196072
Casp7	NM_022260	1.216	0.4659333	0.847	0.6365750	1.122	0.8404701	1.010	0.9095362	1.020	0.9184631
Casp8	NM_022277	1.139	0.6618228	1.056	0.9633907	1.500	0.2629580	0.772	0.4598854	1.412	0.3008877
Casp8ap2	NM_001107921	1.569	0.0023272	1.140	0.2562916	1.243	0.1345891	0.800	0.3165590	0.934	0.9832853
Casp9	NM_031632	1.389	0.1659442	0.914	0.6844373	1.267	0.3695355	0.541	0.0712295	1.225	0.3849312
Cd40	NM_134360	1.018	0.8680653	1.131	0.9411074	1.061	0.9253113	1.100	0.8548994	1.799	0.2610094
Cd40lg	NM_053353	1.103	0.8426040	0.976	0.8519256	0.639	0.3114931	0.953	0.7106299	1.168	0.4135119
Cflar	NM_057138	1.619	0.0050765	1.578	0.0251148	1.337	0.1547765	1.101	0.4886321	0.866	0.4451756
Cidea	NM_001170467	5.769	0.0085289	0.875	0.7748849	1.942	0.1429739	0.634	0.3087657	0.928	0.6239308
Cideb	NM_001108869	1.024	0.9024313	1.200	0.9165045	0.728	0.4010196	1.000	0.7593547	1.458	0.3514201
Cycs	NM_012839	1.370	0.1509402	1.234	0.4931549	0.830	0.3673580	1.226	0.0765290	0.784	0.3949348
Dad1	NM_138910	1.719	0.0124780	1.027	0.7842158	1.416	0.0751706	1.094	0.7592268	0.813	0.6226660
Dapk1	NM_001107335	0.642	0.1629473	0.344	0.0549804	0.610	0.1751841	0.402	0.0616076	0.995	0.8067767
Dffa	NM_053679	0.899	0.4713389	0.623	0.1013463	1.003	0.8879502	0.690	0.0680187	0.917	0.5944969
Dffb	NM_053362	1.113	0.8192872	1.078	0.9455287	0.825	0.4692862	0.912	0.6705871	1.203	0.4079572
Diablo	NM_001008292	1.778	0.0309260	1.040	0.9707353	1.407	0.1756648	0.824	0.3841218	0.829	0.4059028
Fadd	NM_152937	1.044	0.9766536	0.893	0.4910697	1.262	0.3471362	0.981	0.8647543	1.259	0.2374101
Faim	NM_080895	2.054	0.0002074	1.112	0.4343309	2.105	0.0278630	0.788	0.1535163	0.900	0.9400134
Fas	NM_139194	0.931	0.9093033	1.021	0.9600208	1.049	0.8546611	1.000	0.8281013	1.228	0.3971057
Faslg	NM_012908	1.114	0.8333828	0.976	0.8519256	0.639	0.3114931	0.944	0.7029124	1.811	0.2721801
Gadd45a	NM_024127	33.934	0.0043849	26.471	0.0000081	2.580	0.0180250	1.786	0.2956835	0.521	0.2760925
Hrk	NM_057130	1.103	0.8426040	0.976	0.8519256	0.639	0.3114931	0.953	0.7106299	1.208	0.4053550
Il10	NM_012854	1.030	0.7215323	1.783	0.0624628	0.884	0.9685639	1.462	0.4720045	2.050	0.1503620
Lta	NM_080769	1.103	0.8426040	0.976	0.8519256	0.639	0.3114931	0.953	0.7106299	1.179	0.4119190
Ltbr	NM_001008315	1.164	0.2950873	1.711	0.0049555	1.646	0.0050895	0.937	0.6301171	1.185	0.4868120
Mapk1	NM_053842	1.220	0.3023403	0.784	0.3067314	0.774	0.2179223	1.002	0.8929405	0.732	0.4515007
Mapk8ip1	NM_053777	1.009	0.9226539	0.885	0.5126220	0.721	0.1143398	0.938	0.5109765	0.712	0.0245349
Mcl1	NM_021846	1.063	0.2460024	1.563	0.0001276	1.121	0.1918443	1.302	0.0377501	0.706	0.0318773
Naip2	XM_226742	1.103	0.8426040	1.191	0.9238886	2.047	0.1665707	0.953	0.7106299	1.797	0.2917199
Nfkb1	XM_342346	1.275	0.0400524	2.013	0.0009646	1.419	0.0391724	1.552	0.0065084	0.779	0.5168242
Nol3	NM_053516	1.911	0.0022463	0.903	0.3924539	1.516	0.0030619	0.855	0.3667156	0.700	0.1080569
Polb	NM_017141	1.320	0.0783345	0.762	0.0778230	1.067	0.5972600	0.876	0.4446716	0.732	0.1665674
Prdx2	NM_017169	1.821	0.0254643	0.899	0.5358978	1.263	0.0810417	1.000	0.8623954	0.792	0.6288237
Prlr	NM_012630	1.103	0.8426040	0.976	0.8519256	0.639	0.3114931	0.953	0.7106299	1.127	0.4193053
Pycard	NM_172322	2.049	0.0001779	1.647	0.0224429	5.740	0.0085197	1.055	0.5886226	2.015	0.0570722
Ripk2	XM_342810	2.737	0.0000236	0.795	0.3761186	1.528	0.1140157	0.821	0.1079924	0.639	0.0077191
Sphk2	NM_001012066	1.569	0.0532055	0.750	0.3048056	1.217	0.6133062	0.774	0.1497402	0.798	0.1797264
Tnf	NM_012675	1.006	0.8747687	1.534	0.3692731	0.708	0.3683797	1.429	0.6814486	1.577	0.3164771
Tnfrsf10b	NM_001108873	1.089	0.8162706	1.915	0.1878127	0.891	0.5551148	0.972	0.7152231	1.194	0.4135156
Tnfrsf11b	NM_012870	0.868	0.9167994	7.637	0.0002704	1.339	0.5840474	6.242	0.0003507	1.856	0.2811675
Tnfrsf1a	NM_013091	1.741	0.0366190	4.714	0.0010418	2.107	0.0163061	1.861	0.0101588	0.647	0.1630276
Tnfrsf1b	NM_130426	1.236	0.3361461	2.884	0.0002015	2.738	0.0115667	1.504	0.1621738	1.703	0.1177998
Tnfsf10	NM_145681	1.489	0.0640165	0.256	0.0121597	0.968	0.8367897	0.841	0.6907212	0.846	0.7400348
Tnfsf12	NM_001001513	1.330	0.0526604	0.785	0.2363447	1.715	0.0245183	0.649	0.0401609	0.861	0.4840473
Tp53	NM_030989	1.823	0.0034677	1.580	0.0876470	1.245	0.2873340	1.355	0.0876393	0.709	0.2333269
Tp53bp2	XM_223012	1.138	0.1321477	0.533	0.0030354	0.841	0.1430013	0.723	0.0012144	0.790	0.0123704
Tp63	NM_019221	5.347	0.0001305	0.964	0.9520080	1.230	0.2933686	0.902	0.5167838	0.611	0.0824541
Tp73	NM_001108696	1.103	0.8426040	0.976	0.8519256	0.639	0.3114931	0.953	0.7106299	1.127	0.4193053
Tradd	NM_001100480	1.415	0.0729792	0.956	0.6839678	2.127	0.0134618	0.775	0.2086179	1.261	0.3275444
Traf2	NM_001107815	2.236	0.0001078	0.768	0.0298018	1.168	0.2488497	0.871	0.4102884	0.633	0.0309389
Traf3	NM_001108724	1.085	0.3692448	1.056	0.5542144	1.390	0.0042931	1.116	0.6128156	0.875	0.5080657
Xiap	NM_022231	1.169	0.2053883	1.129	0.5044535	1.210	0.2678817	0.993	0.9824334	0.770	0.0355841

Table S2. Differential expression of stress response genes for muscles for old relative to young rats at baseline and for each age group following SSC exposure relative to non-exposed muscles. Expression which surpassed 1.3-fold regulation (below 0.769 fold change or above 1.3 fold change) with a P value < 0.05 were considered differentially expressed; orange - increased expression, blue – decreased expression. Sample sizes were N = 3 to 6 per group.

	Young		Old		Fold change	
	Mean	SE	Mean	SE	Mean	SE
Bax	0.33%	0.25%	0.81%	0.21%	2.47	0.63
Bcl2l1	0.88%	0.32%	2.04%	0.55%	2.31	0.62
Bcl2l11	0.18%	0.05%	0.23%	0.07%	1.28	0.40
Bclaf1	1.51%	0.84%	1.23%	0.28%	0.82	0.19
Bid	2.98%	0.69%	2.58%	0.25%	0.87	0.08
Bik	0.11%	0.07%	0.15%	0.08%	1.37	0.75
Birc2	0.68%	0.26%	1.06%	0.26%	1.55	0.37
Bnip3l	1.59%	0.43%	1.49%	0.35%	0.94	0.22
Casp3	1.08%	0.43%	1.01%	0.20%	0.94	0.18
Casp9	2.66%	0.70%	2.37%	0.40%	0.89	0.15
Cideb	1.08%	0.48%	1.07%	0.25%	1.00	0.23
Cradd	0.20%	0.07%	0.17%	0.04%	0.85	0.23
Dapk1	0.12%	0.08%	0.27%	0.11%	2.38	0.94
Dffa	13.33%	2.10%	11.64%	1.28%	0.87	0.10
Fadd	1.85%	0.91%	1.88%	0.53%	1.01	0.29
Gadd45a	2.53%	0.91%	2.36%	0.37%	0.93	0.15
Hrk	0.86%	0.16%	2.87%	1.03%	3.34	1.20
Tnfrsf10b	0.38%	0.10%	0.34%	0.12%	0.89	0.32
Tnfrs21	0.05%	0.02%	0.04%	0.01%	0.79	0.19
Tp53	0.11%	0.06%	0.09%	0.08%	0.76	0.67

Table S3. Percent methylation of genes relevant to stress response for non-exposed muscles of young and old rats. Fold change calculated as old value divided by young value. Sample sizes of *N* = 9 to 12 per value.

	6 hours						120 hours					
	Non-exposed		Exposed		Fold change		Non-exposed		Exposed		Fold change	
	Mean	SE	Mean	SE	Mean	SE	Mean	SE	Mean	SE	Mean	SE
Bax	0.03%	0.01%	0.57%	0.18%	19.81	6.19	0.63%	0.50%	0.45%	0.21%	0.72	0.34
Bcl2l1	1.17%	0.59%	2.31%	0.99%	1.97	0.84	0.59%	0.24%	0.34%	0.12%	0.57	0.20
Bcl2l11	0.14%	0.05%	0.14%	0.05%	0.97	0.32	0.22%	0.08%	0.22%	0.05%	1.00	0.24
Bclaf1	1.70%	1.61%	2.24%	1.32%	1.32	0.77	1.32%	0.74%	0.20%	0.14%	0.15	0.11
Bid	3.14%	0.89%	3.51%	1.10%	1.12	0.35	2.83%	1.16%	2.51%	0.75%	0.89	0.26
Bik	0.19%	0.14%	0.73%	0.69%	3.79	3.61	0.02%	0.01%	0.03%	0.02%	1.32	0.71
Birc2	0.75%	0.47%	1.08%	0.61%	1.44	0.82	0.61%	0.30%	0.38%	0.15%	0.61	0.24
Bnip3l	1.45%	0.49%	2.56%	1.12%	1.77	0.78	1.74%	0.75%	0.34%	0.09%	0.20	0.05
Casp3	1.15%	0.77%	1.50%	0.59%	1.31	0.51	1.01%	0.49%	0.76%	0.28%	0.76	0.28
Casp9	2.97%	1.38%	3.28%	1.28%	1.11	0.43	2.35%	0.49%	1.57%	0.52%	0.67	0.22
Cideb	1.01%	0.85%	1.74%	0.97%	1.72	0.97	1.16%	0.44%	0.59%	0.30%	0.51	0.26
Cradd	0.10%	0.04%	0.21%	0.10%	2.08	0.98	0.29%	0.12%	0.08%	0.04%	0.28	0.13
Dapk1	0.04%	0.03%	0.08%	0.06%	1.83	1.31	0.19%	0.16%	0.21%	0.07%	1.15	0.38
Dffa	13.29%	3.42%	13.72%	2.49%	1.03	0.19	13.37%	2.84%	10.97%	1.16%	0.82	0.09
Fadd	2.10%	1.74%	2.81%	1.30%	1.34	0.62	1.60%	0.81%	0.61%	0.16%	0.38	0.10
Gadd45a	2.52%	1.78%	3.04%	1.15%	1.21	0.46	2.53%	0.75%	1.00%	0.33%	0.39	0.13
Hrk	0.74%	0.19%	1.57%	0.94%	2.13	1.28	1.01%	0.32%	1.10%	0.19%	1.09	0.19
Tnfrsf10b	0.38%	0.18%	0.75%	0.43%	1.98	1.13	0.39%	0.12%	0.13%	0.11%	0.32	0.27
Tnfrsf21	0.06%	0.04%	0.08%	0.04%	1.35	0.66	0.04%	0.01%	0.03%	0.01%	0.77	0.16
Tp53	0.10%	0.05%	0.21%	0.20%	2.04	1.95	0.15%	0.14%	0.01%	0.00%	0.09	0.01

Table S4. Percent methylation of genes relevant to stress response for SSC-exposed and non-exposed muscles of young rats. Fold change calculated as exposed value divided by non-exposed value. Sample sizes of *N* = 4 to 6 per value.

	6 hours						120 hours					
	Non-exposed		Exposed		Fold change		Non-exposed		Exposed		Fold change	
	Mean	SE	Mean	SE	Mean	SE	Mean	SE	Mean	SE	Mean	SE
Bax	0.51%	0.22%	0.51%	0.15%	0.99	0.29	1.10%	0.32%	1.58%	0.54%	1.43	0.49
Bcl2l1	1.93%	0.44%	2.00%	0.85%	1.04	0.44	2.14%	1.06%	1.36%	0.96%	0.64	0.45
Bcl2l11	0.21%	0.14%	0.18%	0.09%	0.89	0.41	0.26%	0.07%	0.16%	0.03%	0.61	0.12
Bclaf1	1.74%	0.22%	2.42%	1.24%	1.39	0.71	0.73%	0.44%	1.03%	0.52%	1.42	0.71
Bid	2.43%	0.33%	3.08%	0.80%	1.27	0.33	2.74%	0.39%	3.91%	0.83%	1.43	0.30
Bik	0.14%	0.13%	0.92%	0.89%	6.38	6.13	0.15%	0.11%	0.07%	0.03%	0.46	0.22
Birc2	0.88%	0.31%	0.63%	0.20%	0.72	0.23	1.24%	0.42%	0.98%	0.36%	0.79	0.29
Bnip3l	1.57%	0.40%	2.85%	0.91%	1.81	0.58	1.42%	0.61%	1.23%	0.42%	0.87	0.30
Casp3	0.74%	0.22%	1.33%	0.46%	1.80	0.62	1.29%	0.30%	1.27%	0.42%	0.98	0.32
Casp9	2.31%	0.53%	2.98%	0.64%	1.29	0.28	2.43%	0.66%	2.11%	0.45%	0.87	0.19
Cideb	0.83%	0.20%	2.24%	0.76%	2.71	0.91	1.32%	0.46%	1.09%	0.45%	0.83	0.34
Cradd	0.11%	0.07%	0.19%	0.11%	1.63	0.97	0.22%	0.05%	0.24%	0.11%	1.11	0.49
Dapk1	0.14%	0.11%	0.21%	0.13%	1.45	0.90	0.41%	0.18%	0.49%	0.20%	1.20	0.50
Dffa	10.23%	1.32%	11.03%	1.58%	1.08	0.15	13.05%	2.15%	9.95%	1.91%	0.76	0.15
Fadd	1.68%	0.23%	2.77%	1.31%	1.65	0.78	2.07%	1.08%	1.87%	0.48%	0.90	0.23
Gadd45a	2.20%	0.35%	2.81%	0.71%	1.28	0.32	2.51%	0.68%	2.31%	0.73%	0.92	0.29
Hrk	2.58%	1.88%	0.46%	0.13%	0.15	0.04	2.73%	0.73%	1.75%	0.28%	0.64	0.10
Tnfrsf10b	0.34%	0.10%	0.77%	0.44%	2.26	1.30	0.34%	0.23%	0.69%	0.29%	2.01	0.86
Tnfrsf21	0.02%	0.01%	0.02%	0.00%	0.71	0.18	0.06%	0.02%	0.06%	0.01%	0.98	0.22
Tp53	0.01%	0.00%	0.47%	0.31%	57.09	37.50	0.16%	0.15%	0.01%	0.00%	0.07	0.01

Table S5. Percent methylation of genes relevant to stress response for SSC-exposed and non-exposed muscles of old rats. Fold change calculated as exposed value divided by non-exposed value. Sample sizes of N = 6 per value.
